# The epidemiology and burden of cardiovascular diseases in countries of the Association of Southeast Asian Nations (ASEAN), 1990–2021: findings from the Global Burden of Disease Study 2021

**DOI:** 10.1016/S2468-2667(25)00087-8

**Published:** 2025-05-27

**Authors:** GBD 2021 ASEAN Cardiovascular Diseases Collaborators, Lay Hoon Goh, Bryan Chong, Stephanie C.C. van der Lubbe, Jayanth Jayabaskaran, Srinithy Nagarajan, Jobelle Chia, Catherine O Johnson, Xiaochen Dai, Jose M Valderas, Budi Aji, Kim Abbegail Tan Aldecoa, Syed Mohamed Aljunid, Roshan A Ananda, Geminn Louis Carace Apostol, Hany Ariffin, Yuni Asri, Atif Amin Baig, Amiel Nazer C Bermudez, Catherine Bisignano, Muthia Cenderadewi, Hana Chen, Mayank Dalakoti, Ferry Efendi, Emerito Jose A Aquino Faraon, Nelsensius Klau Fauk, Fernando Barroga Garcia, Arief Hargono, Eka Mishbahatul Marah Has, Faizul Hasan, Simon I Hay, Umar Idris Ibrahim, Muhammad Iqhrammullah, Benni Iskandar, Nahlah Elkudssiah Ismail, Jazlan Jamaluddin, Jost B Jonas, Sivesh Kathir Kamarajah, Yun Jin Kim, Maria Dyah Kurniasari, Asep Kusnali, Christina Yeni Yeni Kustanti, Daphne Teck Ching Lai, Graciella Angelica Lukas, Zheng Feei Ma, Santi Martini, Roy Rillera Marzo, Septi Melisa, Farizal Rizky Muharram, Christopher J L Murray, Kamarul Imran Musa, Firzan Nainu, Gustavo G Nascimento, Aqsha Nur, Sok King Ong, Veincent Christian Filipino Pepito, Thantrira Porntaveetus, Dimas Ria Angga Pribadi, Setyaningrum Rahmawaty, Kadar Ramadhan, Sheena Ramazanu, Debby Syahru Romadlon, Yoseph Leonardo Samodra, Siddharthan Selvaraj, Christianus Heru Setiawan, Shazlin Shaharudin, Vetriselvan Subramaniyan, Desy Sulistiyorini, Zhong Sun, Ingan Ukur Tarigan, Jansje Henny Vera Ticoalu, Narayanaswamy Venketasubramanian, Mugi Wahidin, Anggi Lukman Wicaksana, Matthew Aldo Wijayanto, Angga Wilandika, Yves Miel H Zuniga, Gregory A Roth, Nicholas WS Chew, Marie Ng

## Abstract

**Background:**

The Association of Southeast Asian Nations (ASEAN) has undergone substantial epidemiological changes over the past three decades, characterised by a growing burden of cardiovascular disease. This study provides an epidemiological overview of cardiovascular diseases across ASEAN.

**Methods:**

As part of the Global Burden of Diseases, Injuries, and Risk Factors Study 2021, we assessed the prevalence, mortality, years of life lost, years lived with disability, and disability-adjusted life-years (DALYs) of 12 cardiovascular diseases, stratified by age, sex, and location, for ten ASEAN member states (Brunei, Cambodia, Indonesia, Laos, Malaysia, Myanmar, the Philippines, Singapore, Thailand, and Viet Nam) from 1990 to 2021. We examined the contribution of major risk factors associated with cardiovascular disease. Diverse data sources and meta-analytical modelling techniques were used to synthesise data and generate consistent estimates for each metric.

**Findings:**

In 2021, there were 36·8 million (95% uncertainty interval 34·4–38·8) prevalent cases of cardiovascular disease and 1·66 million (1·51–1·80) cardiovascular disease deaths across ASEAN. The total number of DALYs was 42·4 million (38·4–46·2), making cardiovascular disease the leading cause of disease burden in the region. Compared with 1990, the number of individuals with cardiovascular disease has increased by 148·1% (144·0–152·5), whereas the age-standardised prevalence rate rose by 2·5% (1·4–3·6). The highest age-standardised prevalence rate was in Malaysia, followed by Indonesia. The top three leading cardiovascular diseases with the highest age-standardised prevalence rates were ischaemic heart disease (2070·6 [1831·3–2358·2] per 100 000 people), lower extremity peripheral arterial disease (1380·8 [1189·8–1598·7] per 100 000 people), and stroke (1300·6 [1230·5–1375·4] per 100 000 people). The age-standardised mortality rate was highest in Laos (410·9 deaths [337·2–485·9] per 100 000 people). Most cardiovascular disease burden was attributed to high systolic blood pressure, dietary risks, air pollution, high low-density lipoprotein cholesterol, and tobacco use, with high BMI and high fasting plasma glucose rapidly rising as attributive factors.

**Interpretation:**

Cardiovascular disease remained the leading cause of mortality and morbidity in ASEAN in 2021. The number of individuals with cardiovascular disease is expected to rise with an ageing population and socioeconomic advancement. Given the disparities across ASEAN, interventions must be tailored at all levels to address the needs in prevention, treatment, and management.

**Funding:**

The Gates Foundation.

## Introduction

Cardiovascular diseases are the leading cause of mortality and disability-adjusted life-years (DALYs) worldwide.^1^, ^2^ In 2021, an estimated 19·4 million deaths and 428 million DALYs globally were attributed to cardiovascular disease.^3^ Consistent with the global trend, the Association of Southeast Asian Nations (ASEAN) has been grappling with a substantial burden of cardiovascular disease.

ASEAN is a regional cooperation network, established in 1967, aimed at promoting economic growth, political stability, and social progress for its ten member states—Brunei, Cambodia, Indonesia, Laos, Malaysia, Myanmar, the Philippines, Singapore, Thailand, and Viet Nam. Demographics and economic status vary widely across countries,^4^, ^5^ as do health-care systems. Historically, health-care systems in ASEAN have followed a centralised government model funded through tax revenue.^6^ However, driven by increasing demand on health care, middle-income and high-income countries in the region have shifted toward decentralisation, expanding national health insurance systems and broadening private sector involvement.^6^, ^7^ In resource-scarce countries, such as Cambodia, Laos, and Myanmar, external assistance played a crucial part in health service provision and financing given local financial constraints and political instability.^8^, ^9^, ^10^ As the epidemiological landscape shifts from infectious to non-communicable diseases (NCDs),^6^ ASEAN's health-care systems are facing mounting challenges.^11^

**Research in context**

**Evidence before this study**

We conducted a search on PubMed from database inception to Feb 11, 2025, without imposing language or publication date restrictions, for estimates of the burden of cardiovascular diseases in the Association of Southeast Asian Nations (ASEAN). We used the search terms “Association of Southeast Asian nations”, “ASEAN”, “cardiovascular diseases”, “cause of death”, “cerebrovascular disorders”, “coronary heart disease”, “CVD”, “DALY”, “death”, “disease burden”, “epidemiology”, “ischaemic heart disease”, “morbidity”, “mortality”, “prevalence”, “rheumatic heart disease”, “stroke”, “trends”, and ASEAN country names. Most studies (over 300) were based in a single country, with the largest number of publications from Singapore, followed by Malaysia, the Philippines, Thailand, and Viet Nam. Studies from Brunei and Laos were the most scarce. There were more studies on stroke than any other cardiovascular diseases. Several studies examined heart failure in ASEAN. Most studies focused on prevalence and clinical management of cardiovascular disease risk factors at the country or subpopulation level. There were no reports on trends in prevalence, mortality, and morbidity across different types of cardiovascular diseases at the ASEAN regional level.

**Added value of this study**

As part of the Global Burden of Diseases, Injuries, and Risk Factors Study, this study presents the latest evidence on the prevalence and burden of cardiovascular diseases in ASEAN from 1990 to 2021. Our results reveal the effect of demographic changes on cardiovascular disease prevalence, disparities in burden, and major risk factors at the regional and country level across ASEAN.

**Implications of all the available evidence**

Substantial work remains to combat the growing burden of cardiovascular diseases in ASEAN. Member states should strengthen prevention, expand treatment access, and improve the management of cardiovascular diseases for their people.

Cardiovascular diseases are among the fastest growing NCDs in ASEAN^12^ and have been recognised as a major public health priority over the past decade. The ASEAN Post-2015 Health Development Agenda (APHDA) reasserted the prevention and control of NCDs as a key health priority. Regional frameworks, guidelines, and intervention packages were introduced to address cardiovascular diseases and their risk factors.^13^ As the APHDA 2021–25 plan concludes and the planning of the post-2025 programme approaches, ASEAN has an opportunity to revisit existing cardiovascular disease strategies and redirect future efforts. A comprehensive analysis of the prevalence and burden of cardiovascular disease and its risk factors is crucial for understanding the current status of the disease. As part of the Global Burden of Diseases, Injuries, and Risk Factors Study (GBD) 2021, we examine the trends and current status of 12 types of cardiovascular diseases and their associated risk factors, by age and sex, across ASEAN from 1990 to 2021. This paper was produced as part of the GBD Collaborator Network and in accordance with the GBD Protocol.^14^

## Methods

### Overview

The GBD is an international epidemiological research initiative designed to generate temporally and geographically comparable disease burden estimates. GBD 2021 covered 371 diseases and injuries and 88 risk factors across 204 countries and territories.^1^, ^2^, ^15^ The analysis used diverse data sources and robust methods to produce detailed age-specific, sex-specific, and country-specific estimates on incidence, prevalence, cause-specific deaths, years of life lost (YLLs), years lived with disability (YLDs), and DALYs.^1^, ^2^

Disease prevalence and burden were estimated collectively and separately for 12 cardiovascular disease causes including ischaemic heart disease (IHD), ischaemic stroke, haemorrhagic strokes (intracerebral and subarachnoid), atrial fibrillation and flutter, lower extremity peripheral arterial disease, aortic aneurysm, cardiomyopathy and myocarditis, hypertensive heart disease, endocarditis, rheumatic heart disease, non-rheumatic valvular heart disease, and a category for other cardiovascular disease conditions. Details of disease categorisation are presented in the appendix (p 3).

### Data sources

Causes of death (COD) and mortality data were primarily gathered from vital registration and verbal autopsy records, a method involving a structured interview with household members about symptoms before death. COD data were available in all ASEAN countries except Laos. In Brunei, Indonesia, Malaysia, the Philippines, Singapore, Thailand, and Viet Nam, available data date back to the 1990s, with some extending to the 1980s. Further details are presented in the appendix (pp 4–6). A major quality issue with COD data is the presence of non-specific, implausible, or intermediate causes—referred to as garbage codes. Each data source was assessed for the extent of garbage coding. Data sources in which more than 50% of all deaths were classified under major garbage codes that could not be sufficiently corrected for a specific location-year were excluded from the analysis. Otherwise, corrections were applied using established redistribution methods to reassign appropriate underlying causes based on the International Classification of Diseases (ICD) 9 and 10. Details of the methods are described in the appendix (p 10) and previous publications.^1^, ^16^, ^17^

For non-fatal outcomes, incidence and prevalence data were gathered from multiple sources, including systematic reviews of published scientific literature, unpublished registry data, population-representative health surveys, and health system administrative records. For inclusion in the study, diseases had to be clearly defined to allow for alignment or adjustment to standard case definitions. Data collection methods and sample characteristics had to be explicitly stated, and samples needed to represent the general population rather than specific subgroups (eg, prison populations). Data that did not meet these criteria were excluded. Additional criteria might apply to specific causes. Data sources used for non-fatal outcomes in ASEAN can be found in the appendix (pp 6–8). Further details on data inclusion and exclusion criteria for specific cardiovascular causes can be found in GBD 2021 online methods appendices.^18^ Specific strategies for handling data gaps are available in the appendix (pp 10–11).

### Mortality

Causes of death were determined using the ICD versions 9 and 10. Details of the relevant ICD-9 and ICD-10 codes can be found in the appendix (p 9). Garbage code redistribution was applied to several non-fatal, undefined, or intermediate causes, such as cardiac arrest, heart failure, and hypertension using appropriate methods (appendix p 10). To improve the estimation of the underlying mortality rate, a Bayesian noise reduction algorithm was applied to the death data. Verbal autopsy data were used as the data input for total cardiovascular disease, IHD, and total stroke deaths, but were not used for other cardiovascular disease COD. The final cause-specific mortality estimates by age, sex, location, year, and cause were generated using the GBD Cause of Death Ensemble model.^1^ Further details can be found in the appendix (p 10) and previous publications.^1^ GBD population estimates were used to derive the corresponding age-standardised estimates.^5^

### Prevalence

Prevalence is defined as the total number of individuals with a given cause in a specific population at a designated time. Cardiovascular disease prevalence was estimated at a more detailed level for specific disease sequelae. As part of the data processing step, extensive effort was made to correct for systematic bias in the various sources.^19^ For instance, health facility admission data were adjusted to account for readmission, scarcity of multiple diagnoses records, and scarcity of outpatient admissions. Furthermore, adjustments were made using network meta-analysis conducted through the meta-regression-Bayesian, regularised, trimmed software (MR-BRT),^20^ to account for study-level discrepancies in case definitions or variabilities stemming from measurement errors across studies. These processed data were subsequently synthesised through an established epidemiological state-transition model, disease model-Bayesian meta-regression (DisMod-MR) 2.1, to generate prevalence estimates that are consistent with patterns of disease incidence, remission, and mortality. Details on DisMod–MR and non-fatal disease modelling in general can be found in the appendix (p 10) and previous publications.^2^, ^19^, ^21^

### YLLs, YLDs, and DALYs

The disease burden associated with cardiovascular disease was captured through both fatal and non-fatal components. The fatal component was quantified by YLLs, representing the burden of premature mortality. YLLs were calculated by multiplying the number of deaths at each age by the remaining life expectancy at that age. The non-fatal component was measured by YLDs, reflecting the non-fatal impact of diseases and the magnitude of health loss associated with each outcome. YLDs were calculated by multiplying the prevalence of various health outcomes by their respective disability weights.^2^ The total disease burden, represented by DALYs, was calculated by summing YLLs and YLDs by location, year, age, sex, and cause, thus capturing both the fatal and non-fatal effects of health outcomes. Further details of these metrics can be found in previous publications.^1^, ^2^, ^19^

### Risk factors and attributable burden

GBD 2021 included the analysis of 12 risk factors associated with cardiovascular disease, namely, high systolic blood pressure, high low-density lipoprotein (LDL) cholesterol, high BMI, high fasting plasma glucose, kidney dysfunction, air pollution (ambient particulate matter pollution and household air pollution from solid fuels), temperature (high and low), lead exposure, dietary risks, smoking, high alcohol use, and low physical activity. A comparative risk assessment framework was used to estimate the health impact of various risk factors. Statistical models, such as spatiotemporal Gaussian process regression (ST-GPR) or DisMod-MR 2.1, were applied to estimate population-level exposures. Each risk was assigned outcomes to form risk–outcome pairs based on evidence of associations between risks and diseases. The theoretical minimum risk exposure levels (TMRELs), where disease risk is lowest, were predefined. Relative risks were estimated for each risk–outcome pair following the Burden of Proof approach,^22^ which integrates data from systematic reviews. Population-attributable fractions (PAFs) were subsequently calculated for each risk–outcome pair using estimated population-level exposures, relative risks, and TMRELs. Finally, the disease burden attributable to each risk factor was calculated by multiplying the PAF for the risk–outcome pair by the corresponding disease burden measure of interest. Further details can be found in previous publications.^1^, ^2^

Uncertainties at each analytical step were propagated to subsequent stages to derive the final estimates. Markov Chain Monte Carlo sampling was used in DisMod-MR 2.1 to obtain 500 draws from the posterior distribution, and these draws were used to compute a 95% uncertainty interval (UI) for the estimates.

This study complies with the Guidelines on Accurate and Transparent Health Estimates Reporting (appendix pp 12–13).^23^ Analyses were completed with R version 4.4.1.

### Role of the funding source

The funder of this study had no role in study design, data collection, data analysis, data interpretation, or the writing of the report.

## Results

**Figure 1:**
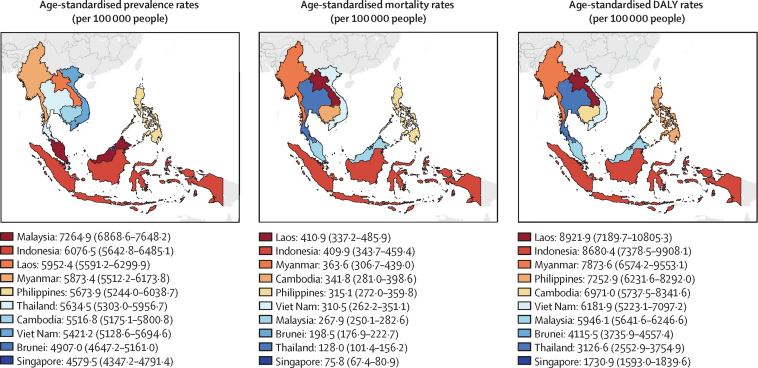
Age-standardised prevalence rates, mortality rates, and DALY rates per 100 000 people for cardiovascular diseases in 2021 DALY=disability-adjusted life-years.

**Figure 2:**
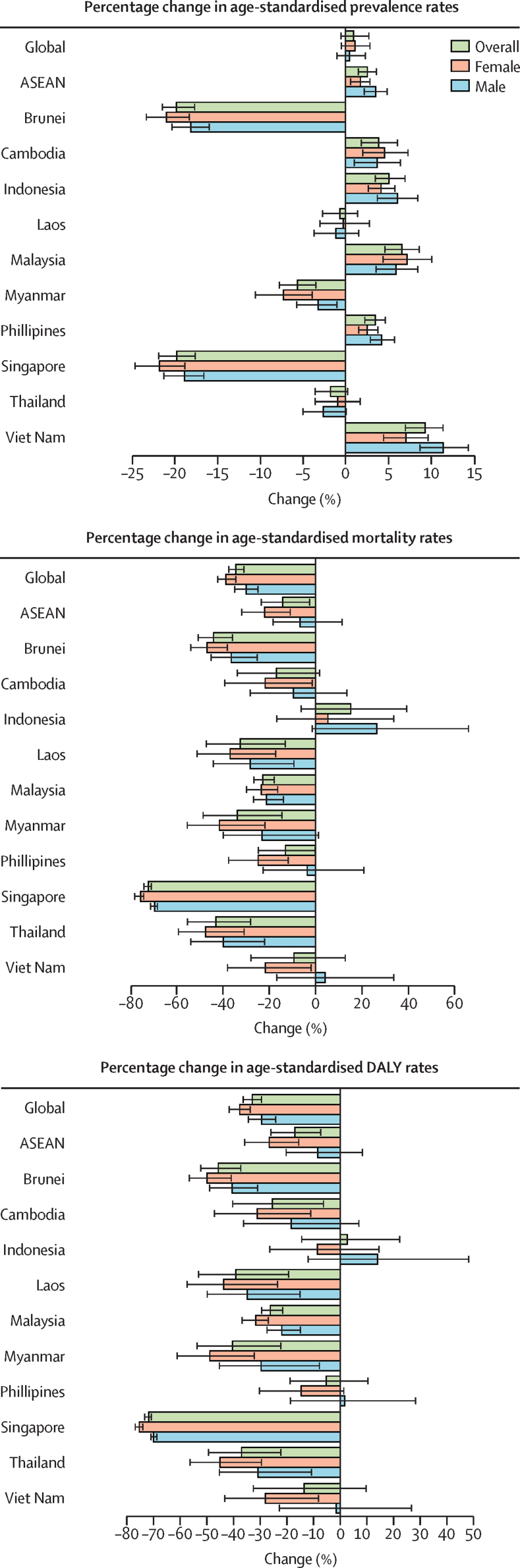
Percentage change in age-standardised prevalence rates, mortality rates, and DALY rates per 100 000 people for cardiovascular diseases from 1990 to 2021 ASEAN=Association of Southeast Asian Nations. DALY=disability-adjusted life-years.

**Figure 3:**
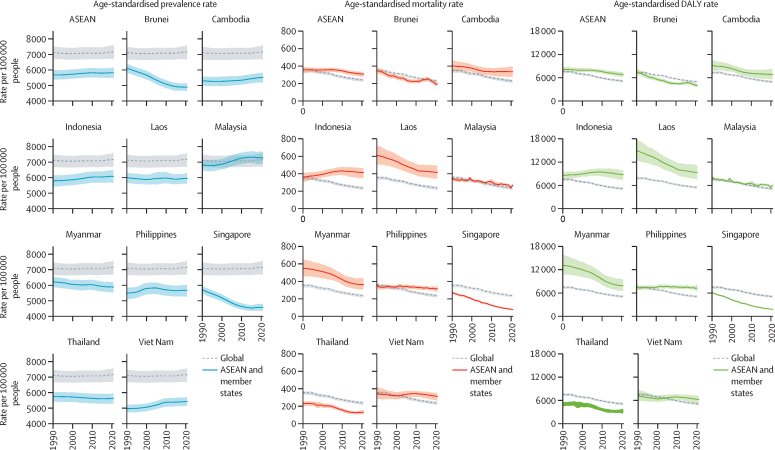
Age-standardised prevalence rates, mortality rates, and DALY rates per 100 000 people for cardiovascular diseases from 1990 to 2021 ASEAN=Association of Southeast Asian Nations. DALY=disability-adjusted life-years.

In 2021, there were 36·8 million (95% UI 34·4–38·8) prevalent cases of cardiovascular diseases in ASEAN (appendix pp 14–15), corresponding to an age-standardised prevalence rate of 5824·5 (5454·3–6144·9) per 100 000 people (appendix p 16). Malaysia had the highest age-standardised cardiovascular disease prevalence rates, estimated at 7264·9 (6868·6–7648·2) per 100 000, followed by Indonesia (6076·5 [5642·8–6485·1] per 100 000) and Laos (5952·4 [5591·2–6299·9] per 100 000; figure 1, appendix p 16). Conversely, the lowest age-standardised prevalence rate was observed in Singapore, with an estimate of 4579·5 (4347·2–4791·4) per 100 000. In general, all countries except Malaysia reported prevalence rates lower than the global average and below those of countries with a similar sociodemographic index. Indonesia's prevalence rate was 1·1 times lower than the middle-SDI average of 6987·0 (6485·0–7482·2).^3^ Similarly, Laos’ prevalence rate was 1·3 times lower than the low-middle SDI average of 7483·4 (6931·3–8031·1).^3^ From 1990 to 2021, the total number of cardiovascular disease cases increased by 148·1% (144·0–152·5; appendix p 17), whereas age-standardised prevalence rates rose by 2·5% (1·4–3·6; figure 2; appendix p 18). Across the ten ASEAN countries, the highest increase in age-standardised prevalence rate was observed in Viet Nam, with a percentage increase in prevalence of 9·2% (7·0–11·4), followed by Malaysia (6·6% [4·5–8·6]), Indonesia (5·0% [3·4–6·9]), Cambodia (3·8% [1·7–6·0]), and the Philippines (3·4% [2·1–4·7]; figures 2, 3; appendix p 18). In contrast, the largest percentage declines in age-standardised prevalence rate were found in Singapore (19·8% decrease [17·7–22·0]) and Brunei (19·8% [17·7–21·5]), followed by Myanmar (5·7% [3·5–7·9]).

In 2021, a total of 1·66 million (95% UI 1·51–1·80) cardiovascular disease deaths were observed across ASEAN (appendix pp 19–20), corresponding to an age-standardised mortality rate of 302·6 (272·9–325·7) per 100 000 population (appendix p 21), making it the leading cause of mortality. Age-standardised mortality rates of cardiovascular diseases in Laos, Indonesia, Myanmar, Cambodia, the Philippines, Viet Nam, and Malaysia, were higher than the global average, with the highest age-standardised mortality rate of 410·9 (337·2–485·9) per 100 000 population in Laos (figure 1; appendix p 21). A similar rate was observed in Indonesia, with an estimate of 409·9 (343·7–459·4) per 100 000 population. Myanmar also recorded a notable rate of 363·6 (306·7–439·0) per 100 000. Brunei (198·5 [176·9–222·7] per 100 000), Thailand (128·0 [101·4–156·2] per 100 000), and Singapore (75·8 [67·4–80·9] per 100 000) observed the lowest age-standardised mortality rates. Compared with countries of similar SDI, Indonesia's age-standardised mortality exceeded that of middle-SDI countries (268·1 per 100 000) by 1·5 times, and the Philippines and Viet Nam both exceeded it by 1·2 times.^3^ Laos exceeded the low-middle SDI average (299·6 per 100 000) by 1·4 times, Myanmar by 1·2 times, and Cambodia by 1·1 times.^3^ Between 1990 and 2021, ASEAN saw an overall increase in the number of deaths of 122·5% (96·7–152·7; appendix p 22) but a decrease in the age-standardised mortality rate of 14·5% (2·3–24·0; appendix p 23). All ASEAN countries except Indonesia observed a reduction in age-standardised cardiovascular disease mortality rates, with the most rapid decreases seen in Singapore (72·4% [71·0–74·4] decrease), Brunei (44·1% [35·6–50·8]), Thailand (43·2% [28·0–55·4]), Myanmar (34·0% [14·3–49·0]), and Laos (32·7% [13·1–47·5]; figures 2, 3; appendix p 23). In contrast, Indonesia saw a 15·1% (–6·4 to 39·6) rise in age-standardised mortality rate.

In ASEAN, cardiovascular diseases contributed to 42·4 million (95% UI 38·4–46·2) DALYs in 2021 (appendix pp 24–25), with an age-standardised DALY rate of 6735·6 (6111·4–7291·6) per 100 000 people (appendix pp 26–27), making it the leading cause of disease burden. The highest age-standardised DALY rates were observed in Laos (8921·9 [7189·7–10 805·3] per 100 000), followed by Indonesia (8680·4 [7378·5–9908·1] per 100 000), and Myanmar (7873·6 [6574·2–9553·1] per 100 000; figure 1; appendix pp 26–27). In contrast, Singapore observed the lowest age-standardised DALY rate of 1730·9 (1593·0–1839·6) per 100 000 people. Thailand (3126·6 [2552·9–3754·9] per 100 000) and Brunei (4115·5 [3735·9–4557·4] per 100 000) also saw relatively low DALY rates. Between 1990 and 2021, the age-standardised DALY rate declined by 17·0% (6·9–26·1) across ASEAN (appendix p 28), whereas the number of DALYs rose by 100·7% (78·3–122·8; appendix p 29). The largest decrease in the age-standardised DALY rate was seen in Singapore (71·9% [70·6–73·3] decline), followed by Brunei (45·8% [36·9–52·4]). Myanmar, Laos, Thailand, Malaysia, and Cambodia also observed declines in age-standardised DALY rates (figure 3; appendix p 28). However, three countries, Indonesia, the Philippines, and Viet Nam, observed relatively constant or no marked changes in age-standardised cardiovascular disease DALY rates over the past 31 years.

Age-standardised prevalence rates were higher in males than in females across ASEAN, except for the Philippines, where the age-standardised cardiovascular disease prevalence rate was higher in females than in males in 2021 (appendix p 34). The largest sex-specific disparities were observed in Singapore and Viet Nam, where age-standardised cardiovascular disease prevalence rates in males were over 1·2 times higher than those in females. In contrast, Indonesia observed the smallest disparities, but both sexes had some of the highest prevalence rates in ASEAN, with estimates of 6107·9 (95% UI 5655·5–6557·4) per 100 000 people in males and 6048·5 (5636·0–6434·9) per 100 000 in females. Age-standardised mortality and DALY rates attributed to cardiovascular diseases were also higher in males than females across ASEAN (appendix pp 35, 36). Singapore and Viet Nam had the largest sex-related disparities in age-standardised mortality (appendix p 35) and DALY rates attributable to cardiovascular diseases (appendix pp 36).

Comparing across age groups, the highest number of cardiovascular disease prevalence cases were observed among individuals aged 65–69 years for both males and females (appendix p 37). However, the number of cardiovascular disease mortality cases peaked earlier in males (aged 70–74 years) than in females (aged 75–79 years). Similarly, the total number of DALYs attributed to cardiovascular diseases peaked earlier for males (aged 60–64 years) than females (aged 70–74 years), suggesting that males have a greater burden of cardiovascular diseases at younger ages than females.

In 2021, the top three leading cardiovascular diseases with the highest age-standardised prevalence rates in the ASEAN region were IHD (2070·6 [95% UI 1831·3–2358·2] per 100 000 people), lower extremity peripheral arterial disease (1380·8 [1189·8–1598·7] per 100 000), and stroke (1300·6 [1230·5–1375·4] per 100 000; appendix p 16). The age-standardised prevalence rates for IHD were the highest in all ASEAN countries except for Brunei, where stroke was the highest (appendix pp 16, 38). Compared with other cardiovascular diseases, pulmonary arterial hypertension (1·9 cases [1·5–2·3] per 100 000) and endocarditis (5·5 [4·8–6·2] per 100 000) had the lowest age-standardised prevalence rates in ASEAN (appendix p 16).

**Figure 4:**
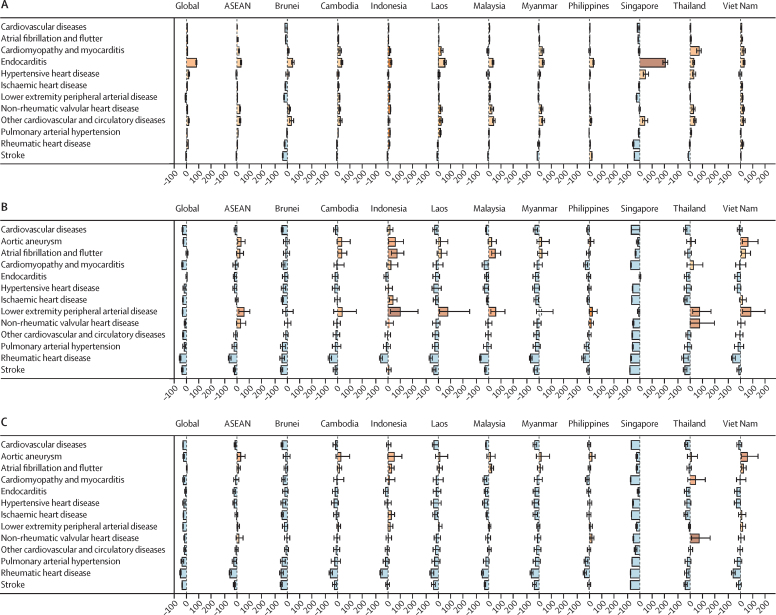
Percentage change in age-standardised (A) prevalence rates, (B) mortality rate, and (C) DALY rates by CVD conditions per 100 000 people from 1990 to 2021 CVD=cardiovascular disease. DALY=disability-adjusted life-years.

From 1990 to 2021 in ASEAN, the largest increase in age-standardised cardiovascular disease prevalence rates was observed for endocarditis, with a percentage increase of 33·0% (95% UI 26·5–41·4), followed by other cardiovascular and circulatory diseases (25·3% [18·7–32·1]) and non-rheumatic valvular heart disease (23·8% [18·8–29·0]; figure 4; appendix p 18). Reductions in age-standardised prevalence were observed for stroke (7·0% [5·6–8·4]) and hypertensive heart disease (2·8% [–9·6 to 3·6]). Singapore observed a marked increase in age-standardised prevalence rate of endocarditis. Although the prevalence rate level and absolute change of endocarditis remained low (5·1 [4·5–5·8] per 100 000 people), the relative percentage increase surpassed the relative changes recorded for other cardiovascular disease subtypes across ASEAN countries (appendix pp 16, 18).

At the ASEAN region level in 2021, stroke had the highest age-standardised mortality rate, estimated at 154·7 (95% UI 139·0–168·8) per 100 000 people, followed by IHD (110·6 [99·8–120·0] per 100 000) and hypertensive heart disease (23·0 [16·7–26·6] per 100 000; appendix p 21). At the country level, however, IHD had the highest age-standardised mortality rates in the Philippines, Malaysia, Brunei, and Singapore, whereas stroke had the highest rates in Indonesia, Viet Nam, Cambodia, Laos, Myanmar, and Thailand.

Although lower extremity peripheral arterial disease (0·1 [95% UI 0·1–0·2] per 100 000 people) and non-rheumatic valvular heart disease (0·4 deaths [0·3–0·6] per 100 000) recorded two of the lowest age-standardised mortality rates in 2021 (appendix p 21), these conditions observed the largest increases in age-standardised mortality rates, with percentage increases of 57·4% (16·6–111·2) in lower extremity peripheral arterial disease and 28·9% (–3·8 to 74·3) in non-rheumatic valvular heart disease, between 1990 and 2021 (figure 4; appendix p 23). During the same period, the largest percentage decreases in age-standardised mortality rates were observed for rheumatic heart disease (65·3% [52·2–73·6]), followed by pulmonary arterial hypertension (24·7% [–41·9 to 0·8]) and hypertensive heart disease (21·5% [–35·1 to 0·6]; appendix p 23).

In terms of DALYs, the highest age-standardised DALY rate was observed for stroke (3446·4 [95% UI 3091·1–3761·3] per 100 000 people), followed by IHD (2413·3 [2177·8–2647·5] per 100 000; appendix pp 26–27). Conversely, pulmonary arterial hypertension, non-rheumatic valvular heart disease, and lower extremity peripheral arterial disease had markedly lower age-standardised DALY rates of fewer than 10 per 100 000 in the ASEAN population. Similar to the findings in mortality rates, IHD in the Philippines, Malaysia, Brunei, and Singapore had the highest age-standardised DALY rates among all cardiovascular disease conditions. However, in Indonesia, Laos, Myanmar, Viet Nam, Cambodia, and Thailand, stroke was the condition with the highest age-standardised DALYs.

From 1990 to 2021, across ASEAN, the only condition with a notable increase in age-standardised DALY rates was aortic aneurysm, with an estimated rise of 31·8% (95% UI 2·0–67·0; appendix p 28). In contrast, four conditions recorded reductions in DALY rates—rheumatic heart disease, pulmonary arterial hypertension, endocarditis, and stroke—with the largest percentage decline of 58·5% (46·0–66·7) observed for rheumatic heart disease (appendix p 28).

**Figure 5:**
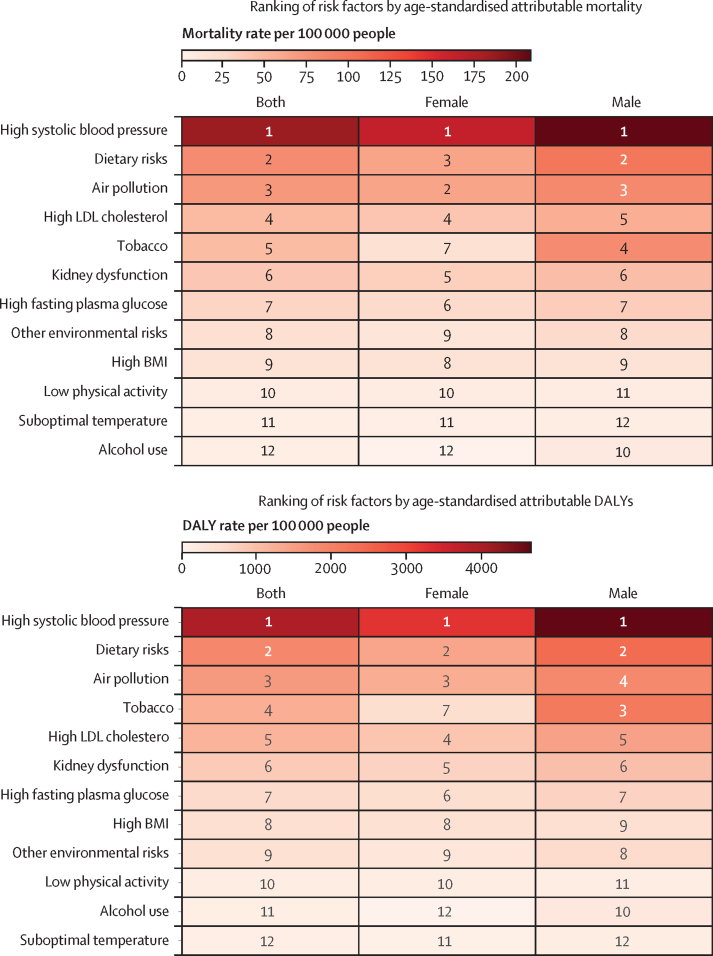
Ranking of risk factors by age-standardised attributable cardiovascular diseases mortality rates and DALY rates per 100 000 people in ASEAN in 2021, by sex ASEAN=Association of Southeast Asian Nations. DALY=disability-adjusted life-years. LDL=low-density lipoproteins.

In 2021, the primary drivers of cardiovascular disease-specific mortality burden were high systolic blood pressure, with an estimated age-standardised mortality rate of 184·8 (95% UI 153·5–212·1) per 100 000, dietary risks (80·4 [18·8–127·0] per 100 000), air pollution (72·8 [54·5– 91·0] per 100 000), high LDL cholesterol (48·1 [26·4–71·1] per 100 000), and tobacco use (47·6 [38·7–57·1] per 100 000) (figure 5; appendix p 30). Similarly, most cardiovascular disease-specific DALYs were attributable to high systolic blood pressure, with an estimated age-standardised DALY rate of 3914·3 (95% UI 3239·4–4534·2) per 100 000 people, followed by dietary risks (1882·5 [389·3–2933·1] per 100 000), air pollution (1572·2 [1181·0–1988·3] per 100 000), tobacco use (1273·0 [1037·5–1508·3] per 100 000), and high LDL cholesterol (1183·6 [709·3–1654·1] per 100 000; figure 4; appendix pp 31–32).

Across all ten ASEAN countries, high systolic blood pressure emerged as the leading risk factor with the highest cardiovascular disease burden in terms of both risk-attributable mortality and DALYs (appendix pp 30–32). Dietary risk was also among the top three risk factors in all countries; however, in Cambodia, Laos, Myanmar, Thailand, and Viet Nam, air pollution contributed slightly more to the cardiovascular disease burden. In Brunei, Malaysia, and Singapore, high LDL cholesterol and high fasting plasma glucose contributed to considerable cardiovascular disease burden. Moreover, tobacco remained a major driver of cardiovascular disease burden, particularly in terms of DALYs, consistently ranking fourth and fifth across all ten countries with respect to attributable DALY rates (appendix pp 31–32).

Between 1990 and 2021, the two risk factors that showed considerable percentage increases in attributable cardiovascular disease-specific burden were high BMI (64·0% [95% UI 24·5–103·4]) and high fasting plasma glucose (15·1% [2·0–31·3]; appendix pp 33, 39). The most substantial rise in cardiovascular disease burden attributable to high BMI was observed in Indonesia, with an estimated percentage increase in age-standardised DALY rate of 112·4% (46·4–200·0), followed by Viet Nam, with an estimated increase of 81·9% (4·0–190·6; appendix p 33). In contrast, five risk factors showed a substantial decline in the associated cardiovascular disease burden—age-standardised cardiovascular disease-specific DALY rates attributable to air pollution decreased by 47·4% (35·3–57·8), dietary risk decreased by 34·5% (22·2–53·9), tobacco use decreased by 22·5% (10·7–32·3), other environmental risks decreased by 21·9% (8·0–32·4), and those related to kidney dysfunction decreased by 12·7% (2·2–22·8). Among ASEAN countries, Singapore showed reductions in age-standardised DALYs across all risk factors (appendix p 39). Brunei and Myanmar also showed declines across most risk factors. In Malaysia and Thailand, although age-standardised DALYs decreased for most risk factors, there was an increase associated with high BMI. In Indonesia and Viet Nam, aside from reductions in DALYs linked to air pollution and dietary risks, changes in DALYs for other causes remained negligible. It is worth noting that sex differences existed in terms of the relative importance of risk factors (figure 5). In males, the top five cardiovascular disease risk factors on attributable age-standardised DALY rates were high systolic blood pressure, dietary risks, tobacco, air pollution, and high LDL cholesterol. In females, the top five cardiovascular disease risk factors on age-standardised DALY rates were high systolic blood pressure, dietary risks, air pollution, high LDL cholesterol, and kidney dysfunction.

## Discussion

This study provides an updated overview of the prevalence and burden of 12 cardiovascular diseases in ASEAN. In 2021, there were an estimated 36·8 million prevalent cases of cardiovascular diseases in ASEAN, accounting for nearly 10% of total cases globally.^19^ The number of prevalent cases across all ages has more than doubled since 1990, and age-standardised prevalence rates observed modest changes, highlighting demographic shifts and population growth as major drivers of cardiovascular diseases in ASEAN. ASEAN observed a total of 1·66 million cardiovascular disease deaths and 42·4 million DALYs in 2021. However, mortality rates differed substantially across countries, ranging from the highest at 410·9 per 100 000 people in Laos to the lowest at 75·8 per 100 000 in Singapore. IHD, lower extremity peripheral arterial disease, and stroke were the most common cardiovascular diseases in 2021. High systolic blood pressure, dietary risks, and air pollution were leading risk factors for cardiovascular disease burden in 2021. However, the fastest-rising risk factor was high BMI, showing a surge of 64·0% in cardiovascular disease burden since 1990.

Discrepancies between cardiovascular disease prevalence and mortality rates in ASEAN exposed the divide in health-care access and quality. In 2021, cardiovascular disease prevalence rates in ASEAN—except Malaysia—were lower than the global average and those of countries with comparable SDI. Yet, mortality rates in most ASEAN countries exceeded both the global average and those of comparable SDI nations. Age-standardised cardiovascular disease mortality rates in Laos and Indonesia were more than 1·7 times higher than the global estimate for 2021. The shortage of life-saving interventions is a major cause of high mortality in this region.^24^, ^25^, ^26^ Equitable access and financing present another major challenge.^27^ The predominance of private institutions in the provision of advanced interventions,^24^ coupled with insufficient public funding^28^ and deficiencies in resource allocation,^29^ aggravates health-care inequalities. The standard of cardiovascular disease care in ASEAN lags international guidelines. Catheter ablation, a preferred intervention for some types of atrial fibrillation, is available only in Singapore and Thailand,^30^ and access remains scarce elsewhere. Cardiac implantable devices, a standard treatment option in high-income nations, are largely inaccessible in much of ASEAN.^31^ The shortage of specialists further constrains treatment delivery.^32^ In 2024, cardiothoracic surgeon density ranged from below 0·01 per 100 000 in Cambodia and Myanmar to 0·7 per 100 000 in Singapore,^33^, ^34^ far below European and US levels.^35^, ^36^, ^37^ At the country level, infrastructure upgrade and development will demand extensive investment in countries such as Cambodia, Myanmar, and Laos.^38^ In the Philippines and Malaysia, equitable access hinges on harmonising public–private sector coordination.^39^, ^40^ At the regional level, ASEAN plays a crucial role in bridging resource-rich and resource-scarce countries. ASEAN's 2009 Mutual Recognition Arrangements on Medical Practitioners, which aimed to enhance mobility, capacity building, and information exchange, can be further leveraged to bolster specialised health-care workforces.^41^ Without closing intervention access gaps, premature mortality from treatable cardiovascular disease conditions could persist, deepening health disparities and hindering ASEAN's socioeconomic progress.^42^

Prioritising prevention, both primary and secondary, is crucial to reducing the burden and economic impact of cardiovascular diseases in ASEAN.^43^ The economic burden of cardiovascular diseases in ASEAN is substantial, ranging from an estimated US$202 million in Cambodia (2018)^44^ to $19 billion in Thailand (2019).^45^ Prevention is pivotal in halting the escalating number of cardiovascular disease cases and reducing health-care costs.^46^ Among primary prevention strategies, tobacco control deserves greater focus in ASEAN.^47^ In 2021, tobacco smoking constituted the third highest cardiovascular disease DALY rate. Scaling up tobacco control is especially pertinent to Indonesia, the only country in the region that has not yet ratified the WHO Framework Convention on Tobacco Control.^48^ For other ASEAN countries, the implementation of tobacco control has yielded measurable success; however, some of these health gains have been offset by the rise of other risk factors, particularly the increase in obesity. The prevalence of obesity has doubled across ASEAN and, in some countries, quadrupled.^49^ Commercial determinants have an important role in driving some of the cardiovascular disease risk factors.^50^ Although local and national political are requisites for policy reform, strong regional commitments are essential. ASEAN, as a collaborative network, must strengthen regulations and adopt trade policies that prioritise public health over corporate interests.^51^

Our analysis also suggested that high systolic blood pressure, hyperlipidaemia, and high fasting plasma glucose are major contributors to the cardiovascular disease burden in ASEAN. One of the most cost-effective intervention for these metabolic risk factors is drug therapies.^52^ However, access to these essential medicines^52^ remains challenging in countries such as Laos,^53^ Cambodia,^54^ and the Philippines.^55^ In the face of the rising cardiovascular disease prevalent cases, ASEAN must capitalise on its established pharmaceutical collaboration structure and devise complementary measures that can ensure equitable access to medicines for all.^56^

Beyond the behavioural and metabolic factors, another leading cardiovascular disease risk factor in ASEAN is air pollution. Over the past decade, driven by rapid urbanisation and industrialisation, the concentration of particulate matter has increased markedly, despite recent signs of reversal.^57^, ^58^ At the turn of the century, WHO pinpointed air pollution as a leading risk factor for cardiovascular disease mortality and morbidity, projecting that two-thirds of these losses would occur in the rapidly developing ASEAN.^59^, ^60^ Transboundary air pollution is an important issue in this region.^61^ The ASEAN Agreement on Transboundary Haze Pollution was intended to address this problem; however, the lack of enforceable provisions has undermined the agreement's impact.^62^ The public health and economic ramifications of air pollution are far-reaching. ASEAN nations must look beyond short-term economic gains and strive for common ground to maintain healthy air quality for their populations.

Consistent with the global trends,^19^ male individuals in ASEAN bore a disproportionate share of the cardiovascular disease burden. Males are more prone to harmful behaviours such as smoking, alcohol consumption, and poor dietary habits compared with female individuals.^63^ Conversely, sex-specific protective factors, such as endogenous oestrogens, might explain the lower atherosclerotic cardiovascular disease risk in females.^64^, ^65^ The largest sex disparities were observed in Singapore and Viet Nam. In Singapore, overweight and obesity were 1·3 times more common in males than in females.^49^ In Viet Nam, males had faster increases in systolic blood pressure and BMI than females,^66^ along with widespread alcohol consumption issues.^67^ In Indonesia, cardiovascular disease prevalence gaps between males and females were narrower, despite higher male smoking rates, possibly due to a greater burden of metabolic syndrome in female individuals.^68^ However, the higher prevalence and burden in males should not be construed as cardiovascular diseases being less important in females. In fact, cardiovascular diseases are the leading cause of mortality and morbidity among females in ASEAN. Addressing this issue requires comprehensive reforms that extend beyond guaranteeing health coverage to tackle entrenched gender inequities.^69^

When interpreting the results of this study, some limitations should be considered. First, the precision of our estimates depends on the availability and quality of primary data. In countries where data are limited or unavailable, estimates are derived from modelling techniques leveraging relevant covariates, as well as spatial and temporal correlation. Interpolated estimates are subject to greater uncertainty. ASEAN must invest in monitoring and surveillance systems to improve health data intelligence, enabling evidence-based decision making.^70^ Second, mortality estimates, primarily based on cause-of-death and verbal autopsy data, are subject to quality issues in settings where vital registrations are incomplete, and ICD coding is not consistently used. The presence of comorbidities at the time of death further complicates the determination of the cause. Although specific correction procedures were applied in the GBD to improve the plausibility of cause assignment, these corrections might have limitations. Third, despite efforts to account for uncertainty in estimates, some measurement or modelling errors might not be fully captured. Fourth, the analysis of cardiovascular disease risk factors relied on relative risk data, which are more readily available from resource-rich countries than resource-poor ones. Therefore, the relative risk estimates applied for the assessment might not reflect potential heterogeneity in risk across countries and populations. The risk factors included in the GBD are also limited to those with sufficient empirical merits in the Burden of Proof analysis.^22^ Other cardiovascular disease risk factors that might show biologically plausible associations with cardiovascular diseases but have not shown strong consistent correlations were not considered. Finally, imperfection exists in the redistribution of garbage codes. Although multiple validation strategies were adopted to ensure rigour, some degree of error remains.

A decade after commissioning the 2015 APHDA, ASEAN has improved in cardiovascular health. However, our study cautions that this progress is countered by an ageing population and the obesity crisis. This situation demands immediate actions focusing on the prevention of cardiovascular disease risk factors, in conjunction with strategic investments to strengthen health systems, and ensuring equitable access to essential cardiovascular disease treatment and management for all. Collaborative efforts among ASEAN member states are vital to overcoming many of the existing challenges. Failure to act could jeopardise ASEAN's health advancement and undermine regional socioeconomic development.

### GBD 2021 ASEAN Cardiovascular Diseases Collaborators

### Affiliations

### Contributors

### Data sharing

To download the input data used and estimates generated in these analyses, please visit the Global Health Data Exchange GBD 2021 (https://ghdx.healthdata.org/gbd-2021). Codes used for the analysis can be found here: https://ghdx.healthdata.org/gbd-2021/code.

## Declaration of interests

NEI reports leadership or fiduciary roles in other board, society, committee or advocacy groups, unpaid as the Bursar and Council Member of the Malaysian Academy of Pharmacy and as a Committee Member of Education Chapter of the Malaysian Pharmacists Society outside the submitted work. VCFP reports grants or contracts from Sanofi Consumer Healthcare to conduct research on self-care in the Philippines, and Bloomberg Foundation through Vital Strategies to conduct research on smoking cessation in the Philippines, and payment or honoraria for lectures, presentations, speakers bureaus, manuscript writing or educational events from the Zuellig Family Foundation outside the submitted work. YLS reports grants or contracts from the Institute of Epidemiology and Preventive Medicine, National Taiwan University; Leadership or fiduciary roles in other board, society, committee or advocacy groups, paid or unpaid as a co-founder of Benang Merah Research Center; and other financial or non-financial interests as a mentor outside the submitted work. JHVT reports leadership or fiduciary roles in other board, society, committee or advocacy groups, paid or unpaid as a co-founder of Benang Merah Research Center outside the submitted work.
