# Supplementary material for: The epidemiology and burden of cardiovascular diseases in countries of the Association of Southeast Asian Nations (ASEAN), 1990–2021: findings from the Global Burden of Disease Study 2021
Source: Lancet Public Health. 2025 May 27;10(6):e467–79. doi: 10.1016/S2468-2667(25)00087-8 (PMC12127266; doi:10.1016/S2468-2667(25)00087-8)
Supplement: Supplementary appendix [file mmc1.pdf]

THE LANCET
Public Health

Supplementary appendix

This appendix formed part of the original submission and has been peer reviewed. We post it as supplied by the authors.

Supplement to: GBD 2021 ASEAN Cardiovascular Diseases Collaborators. The epidemiology and burden of cardiovascular diseases in countries of the Association of Southeast Asian Nations (ASEAN), 1990–2021: findings from the Global Burden of Disease Study 2021. *Lancet Public Health* 2025; **10:** e467–79.

Appendix

Supplement to: The epidemiology and burden of cardiovascular diseases in countries of the Association of Southeast Asian Nations (ASEAN), 1990-2021: findings from the Global Burden of Disease Study 2021

Contents

Appendix 1
GBD CVD disease definition 3
Data sources 4
ICD mapping for CVD causes 9
Statistical methods 10
Garbage codes redistribution 10
Cause of Death Ensemble model (CODEm) 10
Disease model—Bayesian meta-regression (DisMod-MR) 2.1 10
Data gaps and interpolation strategies 10
GATHER checklist 12
Supplementary results 14
References 40
Authors' affiliations 41
Authors' contributions 44

GBD CVD disease definition

The GBD cause list is a hierarchical, mutually exclusive, and collectively exhaustive classification of causes of death. The three Level 1 GBD causes encompass communicable, maternal, neonatal, and nutritional disorders; non-communicable diseases; and injuries. Level 2 causes are divided into 21 groups, such as neoplasms and CVD. Levels 3 and 4 include more specific subcauses. The categorisation of CVD causes is presented in Table S1.

Table S1 GBD CVD disease categorization

Cause Name	Parent Name	Level
**Cardiovascular diseases**	Non-communicable diseases	2
**Rheumatic heart disease**	Cardiovascular diseases	3
**Lower extremity peripheral arterial disease**	Cardiovascular diseases	3
**Endocarditis**	Cardiovascular diseases	3
**Other cardiovascular and circulatory diseases**	Cardiovascular diseases	3
**Ischaemic heart disease**	Cardiovascular diseases	3
**Stroke**	Cardiovascular diseases	3
**Hypertensive heart disease**	Cardiovascular diseases	3
**Non-rheumatic valvular heart disease**	Cardiovascular diseases	3
**Cardiomyopathy and myocarditis**	Cardiovascular diseases	3
**Pulmonary arterial hypertension**	Cardiovascular diseases	3
**Atrial fibrillation and flutter**	Cardiovascular diseases	3
**Aortic aneurysm**	Cardiovascular diseases	3
**Ischaemic stroke**	Stroke	4
**Intracerebral haemorrhage**	Stroke	4
**Subarachnoid haemorrhage**	Stroke	4
**Non-rheumatic calcific aortic valve disease**	Non-rheumatic valvular heart disease	4
**Non-rheumatic degenerative mitral valve disease**	Non-rheumatic valvular heart disease	4
**Other non-rheumatic valve diseases**	Non-rheumatic valvular heart disease	4
**Myocarditis**	Cardiomyopathy and myocarditis	4
**Alcoholic cardiomyopathy**	Cardiomyopathy and myocarditis	4
**Other cardiomyopathy**	Cardiomyopathy and myocarditis	4

Data sources

Table S2 Data sources for causes of death for the ten ASEAN countries

Citation	Country	Year
Brunei Darussalam Vital Registration - Deaths 2017-2018 ICD10. as it appears in WHO Mortality Database Version December 2019	Brunei Darussalam	2017-2018
World Health Organization (WHO). Brunei Darussalam Vital Registration - Deaths 2019 ICD10. as it appears in WHO Mortality Database Version March 2021	Brunei Darussalam	2019
Brunei Vital Registration - Deaths 1996-2010 ICD10. as it appears in WHO Mortality Database Version November 2015	Brunei Darussalam	1996-2010
Brunei Vital Registration - Deaths 2011-2015 ICD10. as it appears in WHO Mortality Database Version November 2017	Brunei Darussalam	2011-2015
Brunei Vital Registration - Deaths 2016 ICD10. as it appears in WHO Mortality Database Version November 2018	Brunei Darussalam	2016
Goyet S, Rammaert B, McCarron M, Khieu V, Fournier I, Kitsutani P, Ly S, Mounts A, Letson WG, Buchy P, Vong S. Mortality in Cambodia: An 18-Month Prospective Community-based Surveillance of All-age Deaths Using Verbal Autopsies. Asia Pac J Public Health. 2013; 1010539513.	Cambodia	2009-2010
Agency of Health Research and Development (Indonesia). Indonesia Basic Health Research 2007-2008.	Indonesia	2007-2008
Ministry of Health (Indonesia). Indonesia Cause of Death Survey 2010-2011.	Indonesia	2010-2011
Agency of Health Research and Development (Indonesia). Indonesia Mortality Registration System Strengthening Project.	Indonesia	2007-2008
Ministry of Health (Indonesia). Indonesia Sample Registration System - Deaths 2012-2014.	Indonesia	2012-2014
Agency of Health Research and Development (Indonesia). Indonesia Sample Registration System - Deaths 2015.	Indonesia	2015
Fortney JA, Susanti I, Gadalla S, Saleh S, Rogers SM, Potts M. Reproductive Mortality in Two Developing Countries. Am J Public Health. 1986; 76(2): 134–8.	Indonesia	1981-1983
Department of Statistics (Malaysia). Vital Statistics: Peninsular Malaysia 1980-1982. Kuala Lumpur, Malaysia: Department of Statistics (Malaysia), 1983-1984.	Malaysia	1980-1982
Malaysia Vital Registration - Deaths 2000-2014 ICD10. as it appears in WHO Mortality Database Version October 2017	Malaysia	2000-2014
Myint, S, Ministry of Health (Myanmar). Cause of Death Verification Study in Myanmar. Presentation at: World Health Organization Regional Office for South East Asia. Regional Consultation on Mortality Statistics; 2007; New Delhi, India.	Myanmar	2007
Bloomberg Philanthropies, Central Statistical Office (Myanmar), Melbourne School of Population and Global Health, University of Melbourne, Ministry of Health (Myanmar). Causes of death in Myanmar using verbal autopsies 2017-2018 [Unpublished data].	Myanmar	2017-2018
Ministry of Health (Myanmar). Myanmar National Mortality Survey 2016.	Myanmar	2016
National Statistics Office (Philippines). Philippines Vital Registration - Deaths 2006-2012.	Philippines	2006-2012

Philippines Statistics Authority. Philippines Vital Registration - Deaths 2013-2018.	Philippines	2013-2018
National Statistics Office (Philippines). Philippines Vital Statistics Report 1991-2005. Manila, Philippines: National Statistics Office (Philippines).	Philippines	1991-2005
Lim Y, Low T, Chan S, Teo T, Jang J, Yip N, Kuntjoro I, Tay E, Yip J. Pulmonary arterial hypertension in a multi-ethnic Asian population: Characteristics, survival and mortality predictors from a 14-year follow-up study. Respirol. 2019; 24(2): 162-70.	Singapore	2003-2016
Ministry of Health (Singapore). Singapore Causes of Death 1980-2017.	Singapore	1980-2017
World Health Organization (WHO). Singapore Vital Registration - Deaths 2019 ICD10. as it appears in WHO Mortality Database Version March 2021	Singapore	2019
Tocharoenvanich P, Yipintsoi T, Choomalee K, Boonwanno P, Rodklai A. Risk factors for a five-year death in the interASIA-south cohort. J Med Assoc Thai. 2008; 91(4): 471-8.	Thailand	2000-2004
Ministry of Public Health (Thailand). Thailand Burden of Disease and Injuries 1998-1999.	Thailand	1998-1999
Porapakkham Y, Rao C, Pattaraarchachai J, Polprasert W, Vos T, Adair T, Lopez AD. Estimated causes of death in Thailand, 2005: implications for health policy. Popul Health Metr. 2010; 8:14.	Thailand	2005
Thailand Vital Registration - Deaths 2011-2018 ICD10. as it appears in WHO Mortality Database Version October 2017	Thailand	2011-2018
World Health Organization (WHO). Thailand Vital Registration - Deaths 2019 ICD10. as it appears in WHO Mortality Database Version March 2021	Thailand	2019
Huong DL, Minh HV, Byass P. Applying verbal autopsy to determine cause of death in rural Vietnam. Scand J Public Health Suppl. 2003; 62: 19-25.	Viet Nam	1999
Hong TT, Phuong Hoa N, Walker SM, Hill PS, Rao C. Completeness and reliability of mortality data in Viet Nam: Implications for the national routine health management information system. PLoS One. 2018; 13(1): e0190755.	Viet Nam	2014
Hieu DT, Hanenberg R, Vach TH, Vinh DQ, Sokal D. Maternal mortality in Vietnam in 1994-95. Stud Fam Plann. 1999; 30(4): 329-38.	Viet Nam	1994-1995
Hoa NP, Rao C, Hoy DG, Hinh ND, Chuc NT, Ngo DA. Mortality measures from sample-based surveillance: evidence of the epidemiological transition in Viet Nam. Bull World Health Organ. 2012; 90(10): 764-72.	Viet Nam	2009
Ngo AD, Rao C, Hoa NP, Adair T, Chuc NTK. Mortality patterns in Vietnam, 2006: Findings from a national verbal autopsy survey. BMC Res Notes. 2010; 3: 78.	Viet Nam	2006-2007
Huong DL, Van Minh H, Janlert U, Van DD, Byass P. Socio-economic status inequality and major causes of death in adults: a 5-year follow-up study in rural Vietnam. Public Health. 2006; 120(6): 497-504.	Viet Nam	1999-2003
Quyen BTT, Nhung NT, Cuong PV. The causes of deaths in Chililab between 2008-2010 based on verbal autopsy method. Vietnam J Public Health. 2012; 1(1): 24-31.	Viet Nam	2008-2010

Hanoi School of Public Health, Ministry of Health (Vietnam), School of Population Health, University of Queensland (Australia). Vietnam Burden of Disease and Injury Study 2008.	Viet Nam	2008

Table S3 Data sources for non-fatal outcomes for the ten ASEAN countries

Citation	Country	Year
Marijon E, Ou P, Celermajer DS, Ferreira B, Mocumbi AO, Jani D, Paquet C, Jacob S, Sidi D, Jouven X. Prevalence of rheumatic heart disease detected by echocardiographic screening. N Engl J Med. 2007; 357(5): 470-6.	Cambodia	2001-2002
Center for Population and Policy Studies, Gadjah Mada University (Indonesia), RAND Corporation, SurveyMETER. Indonesia Family Life Survey 2007-2008. Santa Monica, United States of America: RAND Corporation.	Indonesia	2007-2008
RAND Corporation, SurveyMETER. Indonesia Family Life Survey 2014-2015. Santa Monica, United States of America: RAND Corporation, 2016.	Indonesia	2014-2015
National Team for the Acceleration of Poverty Reduction (TNP2K) (Indonesia), SurveyMETER, University of Southern California, World Bank. Indonesia Family Life Survey East 2012.	Indonesia	2012
Ministry of Health (Indonesia). Indonesia Integrated Hospital Data 2013. Jakarta, Indonesia: Ministry of Health (Indonesia), 2014.	Indonesia	2013
Setyopranoto I, Bayuangga HF, Panggabean AS, Alifaningdyah S, Lazuardi L, Dewi FST, Malueka RG. Prevalence of Stroke and Associated Risk Factors in Sleman District of Yogyakarta Special Region, Indonesia. Stroke Res Treat. 2019; 2019.	Indonesia	2016
Lao-Luxembourg Heart Institute. Laos Rheumatic Heart Disease Prevalence Survey Data 2007-2009.	Lao PDR	2007-2009
World Health Organization (WHO). Laos World Health Survey 2003.	Lao PDR	2003
Aziz ZA, Lee YYL, Ngah BA, Sidek NN, Looi I, Hanip MR, Basri HB. Acute Stroke Registry Malaysia, 2010-2014: Results from the National Neurology Registry. J Stroke Cerebrovasc Dis. 2015; 24(12): 2701–9.	Malaysia	2010-2014
World Health Organization (WHO). Malaysia World Health Survey 2003. Geneva, Switzerland: World Health Organization (WHO), 2005.	Malaysia	2003
World Health Organization (WHO). Myanmar World Health Survey 2003. Geneva, Switzerland: World Health Organization (WHO), 2005.	Myanmar	2003
Htoon MT, Ngwe T, Tun N, Kyaw MM. Prevalence of Cardiovascular Diseases in Rural Area of Hmawbi and Urban Yangon City. Asia Pac J Public Health. 1992; 6(4): 188-94.	Myanmar	Unable to tell from paper

Philippine Health Insurance Corporation. Philippine Health Insurance Corporation Claims 2013-2016.	Philippines	2013-2016
World Health Organization (WHO). Philippines World Health Survey 2003. Geneva, Switzerland: World Health Organization (WHO), 2005.	Philippines	2003
World Health Organization (WHO). WHO Global Programme for the Prevention of Rheumatic Fever and Rheumatic Heart Disease: Report of a Consultation to Review Progress and Develop Future Activities. Geneva, Switzerland: World Health Organization (WHO), 2000.	Philippines	2997-1998
Mak KH, Kark JD, Chia KS, Sim LL, Foong BH, Ding ZP, Kam R, Chew SK. Ethnic variations in female vulnerability after an acute coronary event. Heart. 2004; 90(6): 621-626.	Singapore	1991-1999
Yap KB, Ng TP, Ong HY. Low prevalence of atrial fibrillation in community-dwelling Chinese aged 55 years or older in Singapore: a population-based study. J Electrocardiol. 2008; 41(2): 94-8.	Singapore	Unable to tell from paper
Venketasubramanian N, Tan LCS, Sahadevan S, Chin JJ, Krishnamoorthy ES, Hong CY, Saw SM. Prevalence of Stroke Among Chinese, Malay, and Indian Singaporeans. Stroke. 2005; 36(3): 551-6.	Singapore	2001-2003
Lim Y, Low T, Chan S, Teo T, Jang J, Yip N, Kuntjoro I, Tay E, Yip J. Pulmonary arterial hypertension in a multi-ethnic Asian population: Characteristics, survival and mortality predictors from a 14-year follow-up study. Respirol. 2019; 24(2): 162-70.	Singapore	2003-2016
Ministry of Health (Singapore). Singapore MediClaims Database - Resident Population Inpatient Hospitalization 1991-2006.	Singapore	1991-2006
Ministry of Health (Singapore). Singapore MediClaims Database - Resident Population Inpatient Hospitalization and Outpatient Counts 2007-2017.	Singapore	2007-2017
National Registry of Diseases Office (NRDO), Ministry of Health (Singapore). Singapore Myocardial Infarction Registry 2007-2015.	Singapore	2007-2015
Yeo SH, Yau WP. Temporal Trends and Predictors of Drug Utilization and Outcomes in First-Ever Stroke Patients: A Population-Based Study Using the Singapore Stroke Registry. CNS Drugs. 2019; 33(8): 791-815.	Singapore	2009-2016
Apiyasawat, Sirin; Tangcharoen, Tarinee; Wisaratapong, Treechada; Yamwong, Sukit; Wiboonpolprasert, Suwit; Sritara, Piyamitr. CHA2DS2-VASc scores predict mortality after hospitalization for atrial fibrillation. Int J Cardiol. 2015; 185: 293-6.	Thailand	2005-2010
Tirschwell D, Ton T, Ly K, Van Ngo Q, Vo T, Pham C, Longstreth W, Fitzpatrick A. A prospective cohort study of stroke characteristics,	Viet Nam	2010-2011

care, and mortality in a hospital stroke registry in Vietnam. BMC Neurol. 2012; 12: 150.		
Yamanashi H, Ngoc M, Huy T, Suzuki M, Tsujino A, Toizumi M, Takahashi K, Thiem V, Anh D, Anh N, Tho L, Maeda T, Cox S, Yoshida L, Ariyoshi K. Population-Based Incidence Rates of First-Ever Stroke in Central Vietnam. PLoS One. 2016; 11(8): e0160665.	Viet Nam	2009-2011
Ministry of Health (Vietnam). Vietnam Hospital Data 2013.	Viet Nam	2013
World Health Organization (WHO). Vietnam World Health Survey 2002-2003. Geneva, Switzerland: World Health Organization (WHO), 2005.	Viet Nam	2002-2003

ICD mapping for CVD causes

Table S4 ICD-9 and ICD-10 codes mapping for CVD causes

Causes	ICD-10	ICD-9
**Cardiovascular diseases**	B33.2, G45-G46.8, I01-I01.9, I02.0, I05-I09.9, I11-I11.9, I20-I25.9, I27.0, I27.2, I28-I28.9, I30-I31.1, I31.8-I37.8, I38-I41.9, I42.1-I42.8, I43-I43.9, I47-I48.9, I51.0-I51.4, I60-I63.9, I65-I66.9, I67.0-I67.3, I67.5-I67.6, I68.0-I68.2, I69.0-I69.3, I70.2-I70.8, I71-I73.9, I77-I83.9, I86-I89.0, I89.9, I98, K75.1	391-391.9, 392.0, 393-398.9, 402-402.9, 410-414.9, 416.0, 417-417.9, 420-423, 423.1-423.9, 424.0-424.3, 424.8, 425.0-425.5, 425.7-425.8, 427.0-427.3, 427.6-427.8, 429.0, 430-435.9, 437.0-437.2, 437.5-437.8, 440.2, 440.4, 441-443.9, 447-454.9, 456, 456.3-457, 457.1, 457.8-457.9, 459, 459.1-459.3
**Rheumatic heart disease**	I01-I01.9, I02.0, I05-I09.9	391-391.9, 392.0, 393-398.9
**Ischaemic heart disease**	I20-I25.9	410-414.9
**Stroke**	G45-G46.8, I60-I63.9, I65-I66.9, I67.0-I67.3, I67.5-I67.6, I68.1-I68.2, I69.0-I69.3	430-435.9, 437.0-437.2, 437.5-437.8
**Ischaemic stroke**	G45-G46.8, I63-I63.9, I65-I66.9, I67.2-I67.3, I67.5-I67.6, I69.3	433-435.9, 437.0-437.1, 437.5-437.8
**Intracerebral haemorrhage**	I61-I62, I62.1-I62.9, I68.1-I68.2, I69.1-I69.2	431-432.9, 437.2
**Subarachnoid haemorrhage**	I60-I60.9, I62.0, I67.0-I67.1, I69.0	430-430.9
**Hypertensive heart disease**	I11-I11.9	402-402.9
**Non-rheumatic valvular heart disease**	I34-I37.8	424.0-424.3, 424.8
**Non-rheumatic calcific aortic valve disease**	I35-I35.9	424.1
**Non-rheumatic degenerative mitral valve disease**	I34-I34.9	424.0
**Other non-rheumatic valve diseases**	I36-I37.8	424.2-424.3, 424.8
**Cardiomyopathy and myocarditis**	B33.2, I40-I41.9, I42.1-I42.8, I43-I43.9, I51.4	422-422.9, 425.0-425.5, 425.7-425.8, 429.0
**Myocarditis**	B33.2, I40-I41.9, I51.4	422-422.9
**Alcoholic cardiomyopathy**	I42.6	425.5
**Other cardiomyopathy**	I42.1-I42.5, I42.7-I42.8, I43-I43.9	425.0-425.4, 425.7-425.8, 429.0
**Pulmonary arterial hypertension**	I27.0	416.0
**Atrial fibrillation and flutter**	I48-I48.9	427.3
**Aortic aneurysm**	I71-I71.9	441-441.9
**Lower extremity peripheral arterial disease**	I70.2-I70.8, I73-I73.9	440.2, 440.4, 443.0-443.9
**Endocarditis**	I33-I33.9, I38-I39.9	421-421.9
**Other cardiovascular and circulatory diseases**	I27.2, I28-I28.9, I30-I31.1, I31.8-I32.8, I47-I47.9, I51.0-I51.3, I68.0, I72-I72.9, I77-I83.9, I86-I89.0, I89.9, I98, K75.1	417-417.9, 420-420.9, 423, 423.1-423.9, 427.0-427.2, 427.6-427.8, 442-443, 447-454.9, 456, 456.3-457, 457.1, 457.8-457.9, 459, 459.1-459.3

Statistical methods

Garbage codes redistribution

Garbage codes redistribution was applied to several non-fatal, undefined, or intermediate causes such as cardiac arrest, heart failure, and hypertension using appropriate methods. For instance, deaths coded as heart failure were redistributed using a regression model that accounted for the variability in how these codes were used based on age, sex, and location. Deaths from unspecified types of strokes (ICD-10 I64) were distributed based on the ratio of ischaemic, intracerebral, and subarachnoid events within a country's corresponding GBD region. For regions with limited primary mortality data, the global ratio stratified by age was used. Details of garbage code redistribution can be found in previous publications.[1,2]

Cause of Death Ensemble model (CODEm)

CODEm generates cause-specific mortality estimates through a weighted combination of multiple individual predictive models. These individual predictive models, based on linear mixed-effects models and spatiotemporal Gaussian process regression, were developed using log- or logit-transformed dependent variables with cause-relevant covariates. These models were then ranked by predictive validity and combined accordingly. The final model combination was selected based on out-of-sample predictive error. Further details can be found in previous publications.[2]

Disease model—Bayesian meta-regression (DisMod-MR) 2.1

DisMod-MR 2.1 utilises Bayesian meta-regression techniques to integrate epidemiological data from multiple sources. Mixed-effects models were applied to estimate levels and trends while accounting for heterogeneity across data sources and populations. A built-in compartmental model structure ensures coherence among metrics, transitions between different disease states (e.g., incidence, remission, and mortality). The modelling process allows for incorporation of different priors. These included hierarchical priors to account for geographical structure, informative priors to integrate expert knowledge on disease levels and variability, and age-specific priors to capture age-related patterns in certain conditions. Each cause was modelled separately, with all metrics estimates stratified by age, sex, location, and year. Model development involved an iterative process, where the final specification was selected based on cross-validation results, assessing various goodness-of-fit, error statistics, as well as face validity of conclusion.[3]

Data gaps and interpolation strategies

To address data gaps and generate complete time series for each metric by age, sex, year and location, three key mechanisms were employed in the disease modelling process: (1) the incorporation of covariates, (2) the integration of spatiotemporal

relationships, and (3) the implementation of a compartmental model framework (for DisMod-MR 2.1). With respect to covariates, variables with well-established associations with the outcome metrics of interest were utilised across different stages of analysis. These variables included the Healthcare Access and Quality (HAQ) Index,[4] Socio-demographic Index (SDI),[5] systolic blood pressure, smoking prevalence, and body-mass index (BMI). Moreover, as a part of the broader GBD 2021 study, this analysis leveraged the GBD geographical hierarchical structure that accounts for geographical proximity and epidemiological similarities. Temporal associations were incorporated in the model specifications to capture changes over time. The compartmental model embedded in DisMod-MR 2.1 facilitated interpolations of data by enforcing a coherent mathematical structure. Further details on the estimation process can be found in previous publications.[2,3,6]

GATHER checklist

Item #	Checklist item	Reported location
**Objectives and funding**		
1	Define the indicator(s), populations (including age, sex, and geographic entities), and time period(s) for which estimates were made.	Main text methods overview, paragraph 1
2	List the funding sources for the work.	Main text method section "role of the funding source"
**Data Inputs**		
For all data inputs from multiple sources that are synthesized as part of the study:		
3	Describe how the data were identified and how the data were accessed.	Main text methods section paragraph 1
4	Specify the inclusion and exclusion criteria. Identify all ad-hoc exclusions.	Inclusion criteria summarized in methods section "Data sources"
5	Provide information on all included data sources and their main characteristics. For each data source used, report reference information or contact name/institution, population represented, data collection method, year(s) of data collection, sex and age range, diagnostic criteria or measurement method, and sample size, as relevant.	Table S2 "Causes of death data sources for ASEAN countries" and table S4 "Non-fatal data sources for ASEAN countries"; citations also given on the GHDx (https://ghdx.healthdata.org/)
6	Identify and describe any categories of input data that have potentially important biases (e.g., based on characteristics listed in item 5).	Data inputs in excel format available on the GHDx (https://ghdx.healthdata.org/)
For data inputs that contribute to the analysis but were not synthesized as part of the study:		
7	Describe and give sources for any other data inputs.	N/A
For all data inputs:		
8	Provide all data inputs in a file format from which data can be efficiently extracted (e.g., a spreadsheet rather than a PDF), including all relevant meta-data listed in item 5. For any data inputs that cannot be shared because of ethical or legal reasons, such as third-party ownership, provide a contact name or the name of the institution that retains the right to the data.	Data inputs in excel format available on the GHDx (https://ghdx.healthdata.org/)
**Data analysis**		
9	Provide a conceptual overview of the data analysis method. A diagram may be helpful.	Main text methods overview
10	Provide a detailed description of all steps of the analysis, including mathematical formulae. This description should cover, as relevant, data cleaning, data pre-processing, data adjustments and weighting of data sources, and mathematical or statistical model(s).	Main text methods section with references to previous publications
11	Describe how candidate models were evaluated and how the final model(s) were selected.	Main text methods section with references to previous publications
12	Provide the results of an evaluation of model performance, if done, as well as the results of any relevant sensitivity analysis.	N/A
13	Describe methods for calculating uncertainty of the estimates. State which sources of uncertainty were, and were not, accounted for in the uncertainty analysis.	Main text methods section paragraph 9

14	State how analytic or statistical source code used to generate estimates can be accessed.	N/A
**Results and Discussion**		
15	Provide published estimates in a file format from which data can be efficiently extracted.	The results can be efficiently extracted at https://vizhub.healthdata.org/gbd-results/
16	Report a quantitative measure of the uncertainty of the estimates (e.g. uncertainty intervals).	UIs given for all findings, including in the text, figures, and tables in the main text and appendix; online viz tools (see information above)
17	Interpret results in light of existing evidence. If updating a previous set of estimates, describe the reasons for changes in estimates.	Main text discussion paragraphs 1-8
18	Discuss limitations of the estimates. Include a discussion of any modelling assumptions or data limitations that affect interpretation of the estimates.	Main text discussion, limitations subsection

Supplementary results

Table S5: Number of prevalence cases of CVD causes in ASEAN and its member countries, 2021.

	Association of Southeast Asian Nations	Indonesia	Thailand	Viet Nam	Philippines	Myanmar	Malaysia	Cambodia	Singapore	Laos	Brunei
Cardiovascular diseases	36 779 815 (34 356 620 - 38 839 323)	14 476 458 (13 434 077 - 15 485 854)	5 719 760 (5 381 062 - 6 032 125)	5 307 546 (5 011 599 - 5 594 693)	4 866 014 (4 486 559 - 5 181 747)	2 897 870 (2 706 131 - 3 059 815)	2 099 438 (1 978 746 - 2 219 409)	708 146 (660 346 - 750 953)	385 549 (365 299 - 405 546)	301 527 (279 559 - 322 080)	17 508 (16 494 - 18 577)
Atrial fibrillation and flutter	3 749 544 (2 972 212 - 4 922 042)	1 474 160 (1 152 713 - 1 922 564)	661 374 (516 881 - 864 805)	581 496 (457 784 - 771 131)	466 916 (369 507 - 610 795)	267 795 (211 909 - 352 206)	174 176 (137 798 - 228 296)	61 483 (48 621 - 81 182)	36 795 (28 982 - 47 620)	23 869 (18 913 - 31 406)	1 479 (1 155 - 1 910)
Cardiomyopathy and myocarditis	194 750 (160 880 - 231 367)	71 923 (58 812 - 86 366)	17 427 (14 256 - 21 011)	39 137 (32 336 - 46 191)	32 439 (26 377 - 39 548)	12 438 (10 317 - 14 903)	13 288 (10 992 - 15 684)	2 879 (2 377 - 3 427)	3 638 (2 925 - 4 332)	1 228 (1 007 - 1 460)	352 (289 - 425)
Endocarditis	33 045 (29 112 - 37 093)	6 936 (5 905 - 8 196)	15 244 (13 043 - 17 729)	3 853 (3 369 - 4 358)	2 644 (2 269 - 3 125)	1 189 (1 027 - 1 357)	2 368 (1 992 - 2 743)	324 (281 - 371)	317 (279 - 359)	156 (131 - 184)	14 (12 - 16)
Hypertensive heart disease	919 940 (737 403 - 1 151 098)	380 544 (298 134 - 479 534)	88 634 (68 950 - 116 757)	182 456 (142 953 - 233 597)	144 189 (115 437 - 178 488)	71 192 (55 944 - 91 130)	15 482 (11 993 - 20 002)	20 955 (16 200 - 26 879)	9 560 (7 224 - 12 371)	6 753 (5 273 - 8 611)	176 (126 - 242)
Ischemic heart disease	12 390 673 (10 816 552 - 14 030 556)	5 158 083 (4 325 307 - 6 144 844)	1 888 691 (1 687 559 - 2 111 283)	1 842 805 (1 661 105 - 2 043 084)	1 451 573 (1 221 224 - 1 716 354)	870 841 (784 390 - 966 707)	770 701 (690 323 - 851 719)	207 124 (185 053 - 231 187)	115 852 (109 921 - 122 776)	81 893 (73 304 - 91 918)	3 110 (2 747 - 3 515)
Lower extremity peripheral arterial disease	8 508 460 (7 272 895 - 9 941 273)	3 349 771 (2 838 188 - 3 948 524)	1 369 001 (1 176 716 - 1 590 478)	1 345 189 (1 149 418 - 1 568 553)	1 095 304 (940 456 - 1 279 930)	638 326 (547 104 - 745 864)	393 754 (335 078 - 454 963)	162 250 (138 112 - 190 704)	91 421 (78 716 - 105 642)	59 090 (50 649 - 70 512)	4 353 (3 774 - 5 073)
Non-rheumatic valvular heart disease	639 747 (566 200 - 730 770)	210 892 (194 285 - 230 374)	136 347 (113 851 - 162 308)	91 583 (76 214 - 110 998)	63 828 (58 887 - 69 544)	42 703 (35 199 - 51 952)	35 414 (30 092 - 42 262)	9 732 (8 059 - 11 709)	43 356 (37 143 - 50 102)	3 867 (3 186 - 4 691)	2 026 (1 776 - 2 310)
Other cardiovascular and circulatory diseases	2 442 500 (1 935 004 - 3 049 813)	749 572 (574 194 - 973 563)	609 658 (464 128 - 780 754)	388 426 (307 568 - 500 334)	209 215 (164 732 - 269 712)	142 609 (112 117 - 181 042)	256 695 (204 848 - 321 536)	32 460 (25 370 - 41 702)	39 153 (29 181 - 52 243)	12 726 (10 123 - 16 252)	1 986 (1 403 - 2 750)
Pulmonary Arterial Hypertension	13 047 (10 559 - 16 263)	4 923 (3 971 - 6 154)	1 892 (1 513 - 2 392)	1 990 (1 591 - 2 491)	2 238 (1 836 - 2 776)	858 (681 - 1 059)	594 (479 - 737)	245 (197 - 301)	171 (137 - 212)	126 (103 - 157)	9 (7 - 11)

Rheumatic heart disease	2 946 895 (2 356 162 - 3 606 372)	604 298 (499 659 - 727 451)	498 060 (397 369 - 609 011)	126 388 (108 234 - 147 515)	741 005 (570 571 - 929 343)	502 713 (390 700 - 626 421)	282 150 (223 542 - 350 123)	121 328 (94 362 - 152 790)	1 995 (1 625 - 2 397)	68 686 (52 426 - 86 469)	273 (228 - 321)
Stroke	8 636 340 (8 179 847 - 9 135 219)	3 942 163 (3 639 488 - 4 286 187)	1 070 132 (1 037 337 - 1 109 028)	1 275 345 (1 237 551 - 1 317 875)	1 051 963 (980 977 - 1 134 429)	597 162 (577 053 - 619 885)	400 837 (388 095 - 415 065)	144 798 (140 058 - 150 569)	83 266 (80 410 - 86 242)	65 533 (63 356 - 67 857)	5 142 (4 991 - 5 320)

Table S6: Age-standardised prevalence rates (per 100 000 population) of CVD causes in ASEAN and its member countries, 2021.

	Association of Southeast Asian Nations	Malaysia	Indonesia	Laos	Myanmar	Philippines	Thailand	Cambodia	Viet Nam	Brunei	Singapore
Cardiovascular diseases	5 824.5 (5 454.3 - 6 144.9)	7 264.9 (6 868.6 - 7 648.2)	6 076.5 (5 642.8 - 6 485.1)	5 952.4 (5 591.2 - 6 299.9)	5 873.4 (5 512.2 - 6 173.8)	5 673.9 (5 244.0 - 6 038.7)	5 634.5 (5 303.0 - 5 956.7)	5 516.8 (5 175.1 - 5 800.8)	5 421.2 (5 128.6 - 5 694.6)	4 907.0 (4 647.2 - 5 161.0)	4 579.5 (4 347.2 - 4 791.4)
Atrial fibrillation and flutter	659.9 (518.3 - 851.8)	673.2 (526.9 - 873.0)	728.2 (569.7 - 942.3)	605.1 (475.9 - 782.3)	614.4 (480.4 - 795.8)	653.7 (513.1 - 843.1)	603.8 (470.5 - 784.5)	578.6 (457.2 - 751.3)	644.9 (502.7 - 844.1)	452.0 (354.8 - 583.0)	430.3 (339.4 - 557.4)
Cardiomyopathy and myocarditis	32.4 (27.0 - 38.6)	47.7 (40.1 - 56.6)	32.1 (26.1 - 38.6)	22.4 (18.7 - 27.4)	26.0 (21.5 - 31.7)	34.9 (28.4 - 42.2)	21.1 (17.6 - 25.2)	22.1 (18.5 - 26.6)	43.0 (35.6 - 51.1)	86.6 (70.8 - 103.9)	66.5 (54.5 - 79.8)
Endocarditis	5.5 (4.8 - 6.2)	7.7 (6.6 - 8.9)	2.8 (2.4 - 3.2)	2.4 (2.0 - 2.7)	2.3 (2.0 - 2.6)	2.6 (2.3 - 3.0)	19.4 (16.6 - 22.2)	2.2 (1.9 - 2.5)	4.0 (3.5 - 4.5)	3.6 (3.1 - 4.2)	5.1 (4.5 - 5.8)
Hypertensive heart disease	162.2 (129.8 - 203.6)	58.9 (45.2 - 76.7)	190.4 (148.5 - 240.8)	170.1 (130.0 - 221.1)	162.7 (126.6 - 208.3)	200.0 (157.7 - 249.9)	82.7 (64.3 - 108.5)	196.7 (153.5 - 254.2)	205.7 (159.8 - 267.4)	57.0 (36.2 - 80.5)	114.5 (87.3 - 147.9)
Ischemic heart disease	2 070.6 (1 831.3 - 2 358.2)	2 857.8 (2 581.7 - 3 143.7)	2 349.9 (1 998.8 - 2 782.4)	1 968.3 (1 773.7 - 2 200.5)	1 927.9 (1 745.9 - 2 132.7)	1 881.9 (1 604.1 - 2 205.6)	1 732.2 (1 551.2 - 1 933.3)	1 828.6 (1 645.6 - 2 037.8)	1 961.3 (1 779.9 - 2 182.1)	937.7 (832.5 - 1 056.2)	1 350.8 (1 280.8 - 1 431.8)
Lower extremity peripheral arterial disease	1 380.8 (1 189.8 - 1 598.7)	1 427.2 (1 220.3 - 1 643.1)	1 459.7 (1 254.6 - 1 691.8)	1 352.4 (1 166.3 - 1 584.8)	1 353.4 (1 173.4 - 1 581.4)	1 398.1 (1 209.3 - 1 619.8)	1 237.2 (1 061.8 - 1 433.8)	1 378.5 (1 181.3 - 1 608.7)	1 392.3 (1 197.5 - 1 618.3)	1 348.8 (1 167.9 - 1 561.9)	1 065.4 (916.3 - 1 226.1)
Non-rheumatic valvular heart disease	112.5 (100.1 - 128.3)	134.3 (114.8 - 161.8)	103.1 (95.6 - 111.9)	99.1 (81.6 - 118.3)	97.7 (80.9 - 118.4)	88.2 (81.7 - 95.9)	124.7 (104.3 - 148.6)	89.7 (74.7 - 108.5)	101.7 (84.8 - 122.2)	652.5 (570.0 - 743.5)	506.1 (434.4 - 582.0)
Other cardiovascular and circulatory diseases	385.8 (307.9 - 481.7)	887.5 (706.9 - 1 108.4)	303.6 (235.0 - 391.5)	238.8 (188.9 - 304.3)	284.3 (224.6 - 359.3)	225.8 (173.2 - 296.0)	624.6 (486.7 - 786.7)	247.2 (193.3 - 319.8)	388.3 (310.6 - 496.1)	459.5 (328.6 - 632.9)	494.7 (379.8 - 638.3)
Pulmonary Arterial Hypertension	1.9 (1.5 - 2.3)	1.9 (1.5 - 2.3)	1.7 (1.4 - 2.1)	2.0 (1.6 - 2.4)	1.6 (1.3 - 1.9)	2.2 (1.8 - 2.7)	2.1 (1.7 - 2.6)	1.6 (1.3 - 1.9)	1.9 (1.5 - 2.3)	2.0 (1.6 - 2.5)	2.2 (1.8 - 2.8)
Rheumatic heart disease	415.4 (332.5 - 508.1)	822.5 (654.3 - 1 013.6)	206.7 (171.8 - 248.4)	889.2 (687.1 - 1 114.0)	861.6 (671.4 - 1 073.3)	626.9 (486.6 - 780.1)	723.7 (568.9 - 892.5)	681.9 (534.7 - 853.7)	124.3 (106.0 - 145.4)	70.1 (58.3 - 83.2)	25.2 (20.9 - 29.8)
Stroke	1 300.6 (1 230.5 - 1 375.4)	1 356.7 (1 312.2 - 1 405.2)	1 526.6 (1 405.3 - 1 662.7)	1 232.5 (1 184.7 - 1 283.3)	1 159.7 (1 119.6 - 1 203.4)	1 158.5 (1 074.2 - 1 257.4)	1 050.2 (1 018.7 - 1 087.0)	1 063.0 (1 023.1 - 1 108.7)	1 233.3 (1 193.6 - 1 277.2)	1 384.8 (1 340.6 - 1 433.9)	1 000.4 (966.6 - 1 035.1)

Table S7: Percentage change in the number of prevalence cases by CVD causes from 1990 to 2021

	Association of Southeast Asian Nations	Brunei	Cambodia	Thailand	Laos	Malaysia	Indonesia	Singapore	Philippines	Myanmar	Viet Nam
Cardiovascular diseases	148.1 (144.0 to 152.5)	154.5 (147.7 to 162.8)	159.7 (152.1 to 167.0)	155.8 (146.9 to 166.2)	117.2 (111.6 to 122.9)	190.8 (183.1 to 198.0)	145.4 (140.5 to 150.5)	199.1 (190.6 to 208.0)	167.3 (162.8 to 172.1)	83.8 (78.5 to 89.4)	162.5 (156.8 to 167.8)
Atrial fibrillation and flutter	183.1 (179.2 to 187.0)	181.8 (167.1 to 199.4)	195.3 (185.2 to 206.9)	274.9 (251.2 to 295.7)	139.0 (126.1 to 149.4)	230.6 (212.9 to 246.5)	163.1 (158.4 to 168.4)	255.1 (234.0 to 273.5)	190.1 (186.5 to 194.4)	126.3 (114.9 to 137.8)	171.4 (157.8 to 185.1)
Cardiomyopathy and myocarditis	117.4 (102.3 to 132.4)	74.9 (56.4 to 97.6)	172.5 (145.2 to 199.8)	212.9 (164.6 to 266.4)	174.3 (147.3 to 203.0)	80.6 (60.5 to 105.4)	102.0 (86.5 to 116.6)	87.6 (64.5 to 113.7)	123.6 (109.6 to 137.4)	135.5 (111.9 to 161.3)	122.4 (102.0 to 148.9)
Endocarditis	121.9 (105.9 to 142.1)	163.3 (138.1 to 192.7)	185.6 (164.5 to 206.8)	113.4 (85.8 to 148.9)	243.9 (211.8 to 277.8)	156.9 (138.3 to 179.9)	110.0 (98.2 to 121.6)	642.7 (566.5 to 730.0)	177.3 (162.4 to 191.9)	120.4 (105.7 to 136.6)	110.9 (95.6 to 127.7)
Hypertensive heart disease	160.2 (142.0 to 177.7)	179.9 (141.4 to 227.8)	189.6 (162.9 to 223.6)	349.3 (291.0 to 417.4)	138.9 (110.9 to 171.9)	183.6 (145.2 to 225.7)	147.2 (127.7 to 164.6)	467.1 (383.8 to 568.1)	187.1 (167.8 to 203.8)	122.9 (99.0 to 152.8)	126.8 (101.3 to 158.0)
Ischemic heart disease	179.1 (170.0 to 188.4)	179.3 (163.3 to 197.1)	200.5 (186.7 to 215.5)	219.3 (201.6 to 237.2)	125.9 (113.6 to 137.8)	246.3 (229.0 to 263.5)	180.0 (166.6 to 193.0)	275.7 (249.9 to 305.1)	169.5 (160.1 to 180.3)	102.0 (92.0 to 113.2)	172.1 (160.2 to 186.1)
Lower extremity peripheral arterial disease	187.4 (183.1 to 191.6)	145.0 (134.5 to 156.6)	227.2 (211.4 to 245.5)	225.9 (210.6 to 243.1)	143.3 (131.7 to 154.9)	239.8 (224.4 to 255.0)	187.2 (181.7 to 192.7)	193.8 (179.8 to 207.9)	189.7 (187.0 to 192.8)	106.2 (96.9 to 115.4)	190.3 (175.0 to 205.2)
Non-rheumatic valvular heart disease	235.4 (223.1 to 248.6)	285.0 (249.7 to 321.4)	235.6 (206.0 to 269.2)	355.9 (311.1 to 416.8)	165.6 (140.3 to 192.5)	312.9 (273.0 to 354.3)	206.9 (199.0 to 214.2)	297.7 (264.1 to 332.3)	209.7 (203.0 to 217.5)	169.6 (147.7 to 197.7)	192.6 (167.5 to 221.7)
Other cardiovascular and circulatory diseases	173.1 (155.0 to 194.6)	266.2 (206.2 to 337.5)	215.3 (184.0 to 247.4)	230.2 (190.2 to 276.0)	174.0 (148.7 to 205.5)	256.4 (222.4 to 290.8)	125.2 (103.1 to 149.0)	321.4 (249.9 to 404.8)	157.0 (135.9 to 178.9)	159.9 (132.1 to 191.6)	169.9 (140.0 to 201.4)
Pulmonary Arterial Hypertension	116.0 (102.2 to 129.5)	134.8 (113.2 to 157.8)	123.4 (105.4 to 139.0)	105.3 (81.1 to 131.2)	150.4 (133.5 to 167.5)	130.7 (113.1 to 148.2)	131.0 (116.6 to 145.6)	138.4 (112.9 to 162.8)	113.8 (103.0 to 123.0)	76.8 (63.3 to 93.6)	106.7 (87.5 to 125.9)
Rheumatic heart disease	64.4 (58.5 to 71.3)	125.3 (106.5 to 146.4)	81.2 (68.6 to 96.1)	10.7 (0.8 to 21.2)	96.3 (81.7 to 111.6)	98.6 (86.1 to 114.4)	87.3 (79.9 to 95.0)	32.7 (12.2 to 52.4)	105.5 (100.7 to 111.5)	37.9 (24.3 to 51.8)	153.8 (133.4 to 174.2)
Stroke	116.5 (112.8 to 120.0)	90.7 (86.2 to 95.2)	134.9 (128.3 to 141.5)	106.9 (101.3 to 112.1)	96.5 (91.1 to 101.7)	146.6 (140.4 to 152.3)	105.5 (99.9 to 111.1)	85.0 (79.9 to 90.4)	204.1 (195.7 to 212.3)	63.9 (60.1 to 68.3)	137.1 (130.6 to 144.8)

Table S8: Percentage change of age-standardised prevalence rates (per 100 000 population) of CVD causes from 1990 to 2021

	Association of Southeast Asian Nations	Brunei	Cambodia	Thailand	Laos	Malaysia	Indonesia	Singapore	Philippines	Myanmar	Viet Nam
Cardiovascular diseases	2.5 (1.4 to 3.6)	-19.8 (-21.5 to -17.7)	3.8 (1.7 to 6.0)	-1.8 (-3.6 to 0.2)	-0.7 (-2.8 to 1.4)	6.6 (4.5 to 8.6)	5.0 (3.4 to 6.9)	-19.8 (-22.0 to -17.7)	3.4 (2.1 to 4.7)	-5.7 (-7.9 to -3.5)	9.2 (7.0 to 11.4)
Atrial fibrillation and flutter	3.5 (2.2 to 5.0)	-15.9 (-19.4 to -11.4)	2.8 (-0.3 to 6.5)	4.6 (0.4 to 8.9)	3.0 (-1.2 to 7.1)	6.6 (2.0 to 10.8)	3.9 (2.4 to 5.7)	-13.3 (-16.5 to -9.7)	0.8 (0.1 to 1.8)	0.1 (-4.0 to 4.7)	10.8 (5.9 to 15.7)
Cardiomyopathy and myocarditis	15.5 (8.3 to 22.8)	6.4 (-2.2 to 15.9)	20.6 (6.1 to 33.2)	74.9 (56.0 to 94.1)	25.2 (13.6 to 39.3)	-7.2 (-16.3 to 3.1)	13.4 (4.6 to 22.5)	-5.8 (-14.6 to 2.9)	0.9 (-6.2 to 8.7)	27.0 (12.2 to 41.8)	26.3 (14.2 to 39.6)
Endocarditis	33.0 (26.5 to 41.4)	45.7 (35.2 to 59.2)	32.8 (23.4 to 42.6)	28.8 (20.7 to 38.7)	55.6 (42.9 to 70.1)	34.5 (26.2 to 44.4)	25.5 (18.2 to 32.8)	213.5 (191.9 to 236.4)	34.5 (27.7 to 41.7)	33.5 (25.2 to 43.0)	29.9 (23.0 to 38.4)
Hypertensive heart disease	-2.8 (-9.6 to 3.6)	-1.8 (-14.7 to 17.1)	1.5 (-8.2 to 13.8)	30.2 (15.2 to 48.6)	3.5 (-8.8 to 17.7)	-7.9 (-20.4 to 6.3)	0.5 (-7.7 to 7.3)	46.1 (27.3 to 74.7)	4.3 (-2.3 to 10.3)	-0.1 (-11.3 to 12.9)	-4.2 (-15.2 to 10.1)
Ischemic heart disease	4.4 (0.8 to 7.7)	-16.2 (-20.9 to -10.8)	7.0 (2.2 to 11.9)	-5.3 (-10.5 to 0.0)	0.3 (-4.9 to 5.2)	10.6 (5.5 to 15.8)	12.7 (7.7 to 18.5)	-7.0 (-13.1 to -0.2)	-5.2 (-8.4 to -1.3)	-7.0 (-11.6 to -2.1)	11.5 (6.6 to 17.4)
Lower extremity peripheral arterial disease	6.5 (4.7 to 7.9)	-28.6 (-31.7 to -25.7)	15.0 (9.2 to 21.1)	-1.6 (-5.7 to 2.5)	5.6 (0.9 to 10.1)	8.6 (3.8 to 13.3)	13.1 (11.1 to 15.0)	-28.9 (-32.3 to -25.8)	2.8 (2.0 to 3.8)	-6.6 (-10.7 to -2.5)	15.3 (9.8 to 20.9)
Non-rheumatic valvular heart disease	23.8 (18.8 to 29.0)	16.0 (4.2 to 28.5)	16.1 (5.1 to 28.5)	27.0 (13.5 to 43.5)	20.0 (8.2 to 32.8)	28.0 (14.9 to 41.5)	20.5 (17.4 to 23.5)	-2.7 (-11.2 to 5.9)	6.7 (4.5 to 9.4)	21.4 (11.3 to 34.7)	22.9 (12.6 to 35.1)
Other cardiovascular and circulatory diseases	25.3 (18.7 to 32.1)	34.6 (16.7 to 56.4)	22.9 (11.4 to 35.8)	37.9 (26.5 to 50.2)	22.4 (10.0 to 35.9)	42.0 (28.8 to 55.1)	7.0 (-1.2 to 16.0)	40.7 (20.2 to 68.1)	11.8 (2.4 to 20.7)	31.5 (18.5 to 47.2)	24.9 (12.2 to 37.9)
Pulmonary Arterial Hypertension	7.4 (5.9 to 9.1)	-3.0 (-7.6 to 1.3)	0.0 (-5.7 to 5.7)	7.8 (3.0 to 13.7)	16.4 (9.5 to 23.7)	-0.6 (-5.8 to 5.1)	16.7 (14.4 to 19.1)	-10.4 (-15.7 to -5.2)	-4.7 (-5.8 to -3.7)	1.3 (-4.7 to 7.4)	1.6 (-3.7 to 7.2)
Rheumatic heart disease	0.8 (-2.3 to 3.6)	-23.7 (-28.9 to -17.8)	-4.6 (-11.0 to 2.4)	-2.5 (-8.4 to 4.0)	-3.6 (-11.0 to 3.6)	1.4 (-4.4 to 7.5)	11.4 (7.6 to 15.2)	-55.8 (-61.8 to -49.1)	5.4 (1.9 to 9.3)	-4.6 (-14.7 to 5.5)	16.3 (7.4 to 26.0)
Stroke	-7.0 (-8.4 to -5.6)	-39.6 (-41.1 to -37.9)	-6.5 (-9.1 to -3.4)	-18.2 (-20.3 to -15.9)	-10.8 (-13.5 to -8.2)	-9.1 (-11.4 to -6.6)	-6.7 (-9.0 to -4.3)	-48.6 (-50.1 to -46.8)	19.6 (16.7 to 22.3)	-15.4 (-17.6 to -13.0)	1.7 (-1.4 to 5.2)

Table S9: Number of deaths attributed to CVD causes in 2021

	Association of Southeast Asian Nations	Indonesia	Viet Nam	Philippines	Myanmar	Thailand	Malaysia	Cambodia	Laos	Singapore	Brunei
Cardiovascular diseases	1 664 046 (1 508 942 - 1 798 533)	765 660 (647 217 - 876 429)	261 099 (218 650 - 297 128)	225 939 (193 234 - 259 965)	152 058 (126 346 - 184 972)	136 761 (108 137 - 167 205)	66 779 (62 583 - 70 162)	33 261 (27 055 - 39 723)	15 701 (12 642 - 19 048)	6 291 (5 606 - 6 712)	496 (443 - 556)
Aortic aneurysm	7 460 (6 563 - 8 568)	1 820 (6 987 - 2 446)	895 (656 - 1 193)	950 (779 - 1 119)	392 (296 - 514)	2 134 (1 640 - 2 755)	971 (812 - 1 159)	74 (47 - 119)	34 (25 - 47)	177 (158 - 191)	13 (11 - 15)
Atrial fibrillation and flutter	21 591 (18 292 - 24 987)	6 987 (5 445 - 8 421)	4 015 (2 990 - 5 030)	2 445 (2 054 - 2 912)	1 630 (1 236 - 2 084)	5 064 (3 744 - 6 440)	931 (786 - 1 069)	279 (217 - 359)	130 (102 - 169)	99 (84 - 108)	12 (10 - 13)
Cardiomyopathy and myocarditis	17 868 (15 477 - 20 547)	7 372 (5 644 - 9 464)	3 337 (2 483 - 4 221)	3 052 (2 106 - 3 549)	1 743 (1 348 - 2 305)	880 (639 - 1 133)	839 (708 - 978)	323 (228 - 442)	159 (116 - 221)	141 (125 - 155)	22 (19 - 26)
Endocarditis	7 244 (5 641 - 10 605)	2 001 (1 208 - 3 637)	775 (426 - 1 487)	810 (564 - 977)	513 (300 - 939)	2 489 (1 845 - 3 367)	427 (320 - 515)	115 (65 - 214)	65 (35 - 116)	44 (38 - 48)	4 (3 - 5)
Hypertensive heart disease	124 344 (89 226 - 144 436)	59 126 (37 285 - 77 008)	18 474 (12 002 - 26 383)	24 791 (20 559 - 29 170)	11 893 (6 219 - 17 280)	3 622 (2 696 - 4 743)	1 442 (1 219 - 1 752)	3 155 (1 934 - 4 209)	1 278 (777 - 1 730)	535 (469 - 583)	28 (22 - 36)
Ischemic heart disease	615 547 (555 804 - 671 557)	276 494 (229 238 - 322 155)	64 415 (52 828 - 76 111)	107 668 (91 778 - 124 077)	56 701 (46 130 - 70 566)	50 625 (39 490 - 62 946)	37 821 (35 175 - 40 188)	11 000 (8 639 - 13 511)	6 700 (5 315 - 8 333)	3 896 (3 497 - 4 151)	227 (200 - 256)
Lower extremity peripheral arterial disease	732 (581 - 932)	262 (160 - 410)	120 (78 - 178)	139 (113 - 169)	57 (39 - 81)	50 (35 - 67)	31 (25 - 37)	8 (3 - 15)	5 (3 - 8)	59 (48 - 66)	1 (1 - 1)
Non-rheumatic valvular heart disease	1 798 (1 411 - 2 962)	388 (231 - 1 032)	171 (96 - 470)	148 (106 - 181)	84 (51 - 232)	760 (517 - 1 052)	148 (126 - 185)	16 (9 - 43)	7 (4 - 20)	67 (57 - 73)	7 (5 - 9)
Other cardiovascular and circulatory diseases	7 443 (6 296 - 11 137)	2 986 (2 192 - 5 143)	992 (649 - 1 755)	988 (830 - 1 153)	754 (531 - 1 330)	790 (613 - 996)	657 (567 - 747)	148 (104 - 240)	76 (50 - 132)	47 (40 - 51)	6 (5 - 8)
Pulmonary Arterial Hypertension	633 (433 - 1 754)	268 (170 - 672)	106 (53 - 332)	70 (48 - 184)	75 (48 - 178)	62 (35 - 282)	19 (11 - 65)	17 (10 - 38)	9 (5 - 20)	6 (5 - 7)	1 (1 - 1)
Rheumatic heart disease	7 282 (5 830 - 9 453)	3 397 (2 431 - 5 014)	845 (603 - 1 170)	1 058 (701 - 1 517)	1 000 (726 - 1 415)	309 (240 - 389)	246 (196 - 292)	226 (150 - 316)	149 (91 - 245)	46 (39 - 51)	5 (4 - 6)

Stroke	852 104 (766 130 - 927 958)	404 559 (338 453 - 464 309)	166 954 (139 508 - 193 351)	83 819 (71 374 - 96 158)	77 215 (61 936 - 95 594)	69 976 (54 312 - 86 359)	23 246 (21 070 - 25 704)	17 903 (14 033 - 21 561)	7 088 (5 666 - 8 753)	1 174 (1 016 - 1 280)	170 (147 - 198)

Table S10: Age-standardised mortality rates (per 100 000 population) of CVD causes in 2021

	Association of Southeast Asian Nations	Laos	Indonesia	Myanmar	Cambodia	Philippines	Viet Nam	Malaysia	Brunei	Thailand	Singapore
Cardiovascular diseases	302.6 (272.9 - 325.7)	410.9 (337.2 - 485.9)	409.9 (343.7 - 459.4)	363.6 (306.7 - 439.0)	341.8 (281.0 - 398.6)	315.1 (272.0 - 359.8)	310.5 (262.2 - 351.1)	267.9 (250.1 - 282.6)	198.5 (176.9 - 222.7)	128.0 (101.4 - 156.2)	75.8 (67.4 - 80.9)
Aortic aneurysm	1.5 (1.3 - 1.7)	0.9 (0.7 - 1.3)	1.0 (0.7 - 1.4)	1.0 (0.7 - 1.3)	0.7 (0.5 - 1.2)	1.4 (1.1 - 1.6)	1.1 (0.8 - 1.4)	4.0 (3.4 - 4.9)	4.8 (4.0 - 5.7)	2.0 (1.5 - 2.6)	2.2 (1.9 - 2.3)
Atrial fibrillation and flutter	5.2 (4.4 - 6.1)	5.1 (4.0 - 6.7)	6.4 (4.8 - 7.8)	5.1 (3.9 - 6.6)	4.3 (3.3 - 5.6)	4.8 (4.0 - 5.8)	5.8 (4.4 - 7.4)	4.9 (4.1 - 5.7)	6.2 (5.2 - 7.4)	4.6 (3.4 - 5.8)	1.2 (1.0 - 1.3)
Cardiomyopathy and myocarditis	3.4 (3.0 - 3.9)	4.3 (3.1 - 5.9)	4.6 (3.6 - 5.7)	4.4 (3.4 - 5.8)	3.5 (2.5 - 4.5)	4.5 (2.9 - 5.3)	4.2 (3.1 - 5.3)	3.4 (2.8 - 4.0)	7.1 (5.9 - 8.4)	0.9 (0.7 - 1.1)	1.8 (1.6 - 2.0)
Endocarditis	1.2 (0.9 - 1.8)	1.2 (0.6 - 2.1)	0.9 (0.5 - 1.6)	1.0 (0.6 - 1.9)	0.9 (0.5 - 1.8)	0.9 (0.7 - 1.1)	0.8 (0.5 - 1.6)	1.5 (1.1 - 1.8)	1.6 (1.2 - 2.0)	2.5 (1.9 - 3.4)	0.5 (0.5 - 0.6)
Hypertensive heart disease	23.0 (16.7 - 26.6)	34.9 (21.8 - 46.5)	32.9 (21.4 - 42.3)	28.8 (15.1 - 41.9)	32.4 (20.3 - 41.7)	35.6 (29.6 - 42.0)	22.6 (14.9 - 31.4)	5.9 (5.0 - 7.2)	14.4 (11.6 - 17.8)	3.3 (2.5 - 4.4)	6.4 (5.6 - 7.0)
Ischemic heart disease	110.6 (99.8 - 120.0)	176.5 (141.7 - 213.3)	143.3 (119.3 - 163.3)	138.2 (113.0 - 171.6)	111.5 (89.2 - 134.6)	150.4 (129.2 - 171.9)	77.0 (63.5 - 90.7)	149.7 (138.2 - 159.7)	84.5 (74.3 - 95.9)	47.1 (36.8 - 58.6)	46.7 (41.8 - 49.9)
Lower extremity peripheral arterial disease	0.1 (0.1 - 0.2)	0.2 (0.1 - 0.2)	0.2 (0.1 - 0.2)	0.1 (0.1 - 0.2)	0.1 (0.0 - 0.2)	0.2 (0.2 - 0.2)	0.1 (0.1 - 0.2)	0.1 (0.1 - 0.2)	0.6 (0.4 - 0.7)	0.0 (0.0 - 0.1)	0.7 (0.6 - 0.8)
Non-rheumatic valvular heart disease	0.4 (0.3 - 0.6)	0.2 (0.1 - 0.6)	0.2 (0.1 - 0.7)	0.2 (0.1 - 0.6)	0.2 (0.1 - 0.5)	0.2 (0.2 - 0.3)	0.2 (0.1 - 0.6)	0.6 (0.5 - 0.8)	3.7 (2.6 - 4.6)	0.7 (0.5 - 1.0)	0.8 (0.7 - 0.9)
Other cardiovascular and circulatory diseases	1.3 (1.1 - 1.9)	1.5 (1.0 - 2.7)	1.4 (1.0 - 2.4)	1.6 (1.1 - 2.8)	1.2 (0.9 - 2.1)	1.1 (1.0 - 1.3)	1.1 (0.7 - 2.0)	2.5 (2.1 - 2.8)	2.2 (1.8 - 2.6)	0.8 (0.6 - 1.0)	0.6 (0.5 - 0.6)
Pulmonary Arterial Hypertension	0.1 (0.1 - 0.3)	0.2 (0.1 - 0.4)	0.1 (0.1 - 0.4)	0.2 (0.1 - 0.4)	0.1 (0.1 - 0.4)	0.1 (0.0 - 0.2)	0.1 (0.1 - 0.4)	0.1 (0.0 - 0.3)	0.3 (0.2 - 0.3)	0.1 (0.0 - 0.3)	0.1 (0.1 - 0.1)
Rheumatic heart disease	1.1 (0.9 - 1.4)	2.3 (1.4 - 3.8)	1.3 (0.9 - 1.9)	1.9 (1.4 - 2.6)	1.6 (1.0 - 2.1)	1.0 (0.7 - 1.5)	0.9 (0.6 - 1.2)	0.8 (0.6 - 1.0)	2.1 (1.7 - 2.5)	0.4 (0.3 - 0.5)	0.6 (0.5 - 0.6)
Stroke	154.7 (139.0 - 168.8)	183.7 (148.5 - 223.8)	217.7 (182.0 - 245.4)	181.1 (147.6 - 222.2)	185.2 (148.0 - 220.1)	114.8 (98.7 - 131.2)	196.6 (164.2 - 226.8)	94.3 (84.5 - 104.8)	71.1 (60.5 - 81.7)	65.6 (51.0 - 80.8)	14.2 (12.3 - 15.6)

Table S11 Percentage change of the number of deaths attributed to CVD causes from 1990 to 2021

	Association of Southeast Asian Nations	Brunei	Cambodia	Thailand	Laos	Malaysia	Indonesia	Singapore	Philippines	Myanmar	Viet Nam
Cardiovascular diseases	122.5 (96.7 to 152.7)	59.8 (39.0 to 88.2)	111.8 (67.4 to 167.8)	105.9 (63.1 to 163.5)	41.2 (7.9 to 87.3)	125.4 (113.6 to 140.0)	154.8 (108.7 to 207.2)	18.1 (8.7 to 24.7)	169.4 (131.0 to 216.0)	35.8 (3.9 to 79.1)	112.0 (64.7 to 168.2)
Aortic aneurysm	258.3 (181.1 to 352.6)	162.7 (98.5 to 263.2)	266.9 (146.1 to 469.5)	290.5 (165.6 to 469.3)	163.5 (73.2 to 314.5)	262.8 (167.4 to 386.3)	266.0 (135.2 to 430.8)	237.1 (202.0 to 267.7)	225.8 (162.9 to 310.0)	160.8 (73.2 to 314.3)	275.6 (158.5 to 483.6)
Atrial fibrillation and flutter	265.5 (188.0 to 352.7)	131.4 (80.7 to 220.5)	274.6 (178.9 to 393.4)	319.9 (198.9 to 486.1)	217.5 (131.2 to 346.8)	293.1 (216.9 to 410.2)	267.9 (167.8 to 384.6)	213.9 (180.9 to 237.4)	253.9 (187.4 to 335.8)	223.5 (124.3 to 355.3)	229.3 (146.8 to 339.6)
Cardiomyopathy and myocarditis	116.5 (74.0 to 186.2)	60.2 (27.0 to 101.6)	131.6 (63.0 to 286.4)	277.8 (134.3 to 497.4)	86.2 (16.6 to 218.2)	63.1 (22.0 to 130.0)	144.5 (76.0 to 246.3)	-20.3 (-28.8 to -12.3)	100.5 (62.5 to 148.4)	75.8 (20.8 to 167.1)	113.8 (39.8 to 231.2)
Endocarditis	71.4 (45.6 to 96.4)	116.5 (59.4 to 188.6)	85.2 (27.8 to 145.7)	80.2 (33.4 to 143.7)	78.0 (19.1 to 153.1)	92.3 (62.0 to 134.8)	64.3 (28.2 to 100.8)	310.3 (261.7 to 349.7)	116.6 (82.0 to 156.8)	20.6 (-19.2 to 74.2)	56.5 (8.5 to 113.6)
Hypertensive heart disease	109.7 (74.2 to 173.2)	68.1 (24.4 to 144.8)	100.6 (38.6 to 203.7)	167.1 (86.8 to 328.2)	33.2 (-10.6 to 179.1)	90.7 (49.4 to 180.9)	130.5 (77.8 to 215.8)	87.0 (67.5 to 101.4)	192.1 (144.4 to 248.7)	33.0 (-5.5 to 124.2)	64.1 (13.2 to 145.3)
Ischemic heart disease	155.4 (123.0 to 190.3)	68.7 (42.1 to 99.9)	147.8 (87.5 to 216.7)	108.4 (59.9 to 168.4)	59.7 (18.8 to 121.0)	152.0 (131.1 to 174.1)	210.6 (148.1 to 285.9)	33.1 (22.6 to 40.5)	173.9 (132.9 to 225.1)	48.5 (10.2 to 104.1)	173.7 (109.0 to 252.1)
Lower extremity peripheral arterial disease	320.5 (205.7 to 477.8)	127.9 (46.8 to 309.0)	268.4 (66.3 to 632.2)	629.2 (381.0 to 1015.0)	281.5 (131.4 to 740.7)	347.5 (226.7 to 577.1)	358.6 (162.0 to 724.8)	353.9 (294.8 to 409.8)	272.7 (191.9 to 420.7)	162.8 (65.0 to 389.6)	340.9 (169.6 to 643.9)
Non-rheumatic valvular heart disease	225.7 (147.0 to 340.3)	157.1 (103.1 to 239.5)	133.9 (60.0 to 231.4)	530.2 (260.4 to 954.6)	79.6 (10.4 to 178.7)	157.9 (104.3 to 248.1)	135.8 (71.4 to 214.1)	98.8 (74.8 to 117.6)	261.1 (200.4 to 325.9)	73.5 (10.1 to 155.7)	137.6 (69.0 to 216.1)
Other cardiovascular and circulatory diseases	83.6 (55.1 to 120.3)	118.1 (69.4 to 182.5)	77.4 (26.3 to 167.3)	100.8 (51.6 to 177.9)	32.7 (-10.0 to 118.6)	152.1 (95.4 to 211.5)	82.0 (37.0 to 131.5)	32.6 (17.8 to 45.4)	102.8 (71.4 to 142.8)	29.1 (-7.9 to 90.3)	94.4 (26.3 to 158.6)
Pulmonary Arterial Hypertension	39.9 (5.7 to 92.1)	8.3 (-20.7 to 66.8)	41.5 (-19.0 to 149.2)	56.8 (-3.6 to 154.4)	15.6 (-27.4 to 101.2)	90.6 (22.1 to 172.4)	44.7 (4.4 to 109.3)	5.2 (-8.3 to 18.1)	35.7 (4.4 to 87.5)	7.6 (-31.8 to 84.9)	51.1 (-8.2 to 148.5)
Rheumatic heart disease	-36.0 (-49.7 to -12.6)	38.3 (-1.1 to 92.9)	-36.1 (-57.7 to -1.8)	-24.6 (-54.4 to 36.7)	-38.2 (-61.9 to 0.7)	-34.7 (-54.5 to -18.3)	-35.2 (-53.7 to -7.2)	-4.0 (-17.8 to 7.2)	-3.3 (-29.2 to 27.1)	-58.0 (-71.0 to -35.0)	-31.0 (-55.8 to 7.2)
Stroke	107.5 (82.2 to 136.2)	38.2 (15.5 to 71.4)	100.3 (54.2 to 152.8)	92.0 (49.5 to 149.5)	29.5 (-4.0 to 69.3)	95.2 (73.4 to 121.2)	135.3 (93.7 to 183.1)	-31.3 (-38.8 to -25.4)	166.2 (128.1 to 211.6)	29.3 (-2.4 to 75.0)	101.2 (55.0 to 154.9)

Table S12: Percentage change of age-standardised mortality rates (per 100 000 population) of CVD causes from 1990 to 2021

	Association of Southeast Asian Nations	Brunei	Cambodia	Thailand	Laos	Malaysia	Indonesia	Singapore	Philippines	Myanmar	Viet Nam
Cardiovascular diseases	-14.5 (-24.0 to -2.3)	-44.1 (-50.8 to -35.6)	-16.9 (-34.2 to 1.8)	-43.2 (-55.4 to -28.0)	-32.7 (-47.5 to -13.1)	-22.8 (-27.1 to -17.7)	15.1 (-6.4 to 39.6)	-72.4 (-74.4 to -71.0)	-13.2 (-24.9 to 0.2)	-34.0 (-49.0 to -14.3)	-9.3 (-28.4 to 13.0)
Aortic aneurysm	34.9 (6.8 to 69.1)	-14.6 (-35.6 to 15.0)	34.8 (-7.5 to 107.2)	-0.4 (-32.8 to 44.4)	18.2 (-18.3 to 79.1)	22.4 (-9.2 to 65.7)	62.2 (4.4 to 130.4)	-18.9 (-26.7 to -11.6)	10.6 (-11.1 to 36.1)	20.8 (-18.4 to 84.7)	60.4 (12.1 to 146.4)
Atrial fibrillation and flutter	23.9 (-2.8 to 53.6)	-13.0 (-32.0 to 19.0)	35.1 (1.3 to 75.6)	-21.5 (-44.4 to 9.7)	22.6 (-9.4 to 73.6)	53.7 (22.9 to 100.0)	76.6 (26.0 to 134.8)	-37.0 (-42.9 to -32.4)	-8.4 (-24.7 to 6.9)	23.8 (-13.4 to 72.0)	40.8 (5.0 to 86.9)
Cardiomyopathy and myocarditis	-15.8 (-34.4 to 11.5)	-36.5 (-50.3 to -20.3)	-6.6 (-31.4 to 55.4)	26.6 (-28.5 to 105.1)	-18.9 (-44.7 to 38.5)	-38.3 (-55.2 to -7.4)	23.7 (-11.5 to 81.3)	-76.6 (-78.8 to -74.4)	-36.5 (-48.1 to -18.1)	-18.2 (-43.6 to 26.1)	-6.9 (-40.8 to 45.1)
Endocarditis	-20.8 (-32.5 to -7.3)	-20.7 (-40.7 to 6.0)	-21.7 (-44.0 to 4.3)	-32.5 (-48.6 to -10.8)	-24.3 (-47.7 to 8.1)	-22.6 (-35.6 to -3.0)	-17.9 (-34.9 to -0.1)	0.9 (-8.9 to 9.7)	-11.1 (-26.4 to 8.1)	-33.2 (-52.6 to -7.3)	-28.1 (-49.5 to -1.2)
Hypertensive heart disease	-21.5 (-35.1 to 0.6)	-36.4 (-51.8 to -14.5)	-24.2 (-45.8 to 12.4)	-31.4 (-51.4 to 8.4)	-38.0 (-56.8 to 24.7)	-36.1 (-50.0 to -5.1)	1.6 (-21.7 to 38.7)	-60.8 (-64.5 to -57.9)	-3.6 (-18.9 to 14.6)	-37.7 (-55.2 to 2.9)	-29.2 (-51.0 to 4.1)
Ischemic heart disease	-2.7 (-14.5 to 10.8)	-43.5 (-52.0 to -33.7)	-3.6 (-26.3 to 20.6)	-44.3 (-57.4 to -27.6)	-25.0 (-43.1 to 0.1)	-15.7 (-22.9 to -7.9)	39.1 (11.2 to 74.2)	-68.5 (-70.7 to -66.8)	-13.6 (-26.0 to 0.3)	-28.5 (-45.6 to -3.9)	16.0 (-10.8 to 48.0)
Lower extremity peripheral arterial disease	57.4 (16.6 to 111.2)	-12.8 (-43.3 to 49.6)	37.8 (-35.2 to 158.3)	79.6 (18.8 to 174.4)	77.3 (10.7 to 264.8)	59.6 (17.0 to 139.2)	104.5 (19.9 to 254.3)	-10.6 (-21.3 to 0.5)	22.1 (-3.1 to 64.9)	21.8 (-23.6 to 119.7)	84.4 (15.1 to 207.5)
Non-rheumatic valvular heart disease	28.9 (-3.8 to 74.3)	-1.1 (-24.2 to 30.3)	-7.5 (-34.5 to 24.7)	78.9 (-0.6 to 203.5)	-19.9 (-47.5 to 14.6)	-8.0 (-27.9 to 25.3)	12.1 (-16.8 to 44.8)	-57.3 (-61.6 to -53.6)	12.8 (-6.8 to 32.6)	-16.9 (-44.0 to 16.0)	3.2 (-26.3 to 35.8)
Other cardiovascular and circulatory diseases	-11.9 (-28.2 to 3.0)	-16.0 (-33.2 to 7.6)	-14.5 (-37.2 to 15.5)	-21.4 (-40.3 to 6.0)	-29.1 (-49.3 to 5.1)	-2.8 (-27.1 to 20.3)	-2.5 (-25.0 to 19.9)	-66.2 (-69.5 to -63.2)	-4.3 (-19.6 to 12.0)	-24.3 (-43.8 to 4.4)	-8.8 (-41.1 to 20.0)
Pulmonary Arterial Hypertension	-24.7 (-41.9 to 0.8)	-43.1 (-57.9 to -17.8)	-20.9 (-46.3 to 26.3)	-24.2 (-48.6 to 14.1)	-33.3 (-54.0 to 2.1)	-16.2 (-46.7 to 19.1)	-15.2 (-37.8 to 20.7)	-61.6 (-65.8 to -57.1)	-29.6 (-46.4 to -7.0)	-30.1 (-50.8 to 9.9)	-21.6 (-51.3 to 25.4)
Rheumatic heart disease	-65.3 (-73.6 to -52.2)	-47.1 (-62.7 to -27.8)	-68.1 (-78.9 to -50.8)	-54.7 (-73.0 to -17.9)	-69.2 (-80.3 to -52.1)	-72.1 (-80.6 to -64.7)	-63.2 (-73.0 to -47.3)	-76.5 (-79.5 to -73.8)	-53.7 (-67.1 to -39.6)	-72.8 (-81.1 to -58.6)	-65.7 (-78.3 to -44.9)
Stroke	-20.6 (-30.2 to -9.3)	-51.1 (-58.5 to -40.6)	-22.1 (-39.4 to -3.3)	-46.1 (-57.9 to -29.7)	-38.2 (-52.9 to -19.9)	-32.7 (-40.1 to -23.6)	5.5 (-14.2 to 26.3)	-84.3 (-85.8 to -83.0)	-14.1 (-25.9 to -0.9)	-37.5 (-52.5 to -16.7)	-14.3 (-33.4 to 7.4)

Table S13: Number of DALYs attributed to CVD causes in 2021

	Association of Southeast Asian Nations	Indonesia	Philippines	Viet Nam	Myanmar	Thailand	Malaysia	Cambodia	Laos	Singapore	Brunei
Cardiovascular diseases	42 425 108 (38 429 432 - 46 234 629)	20 308 893 (17 200 208 - 23 640 718)	6 187 192 (5 285 912 - 7 116 674)	5 861 342 (4 880 090 - 6 800 537)	3 788 555 (3 123 493 - 4 621 806)	3 185 944 (2 592 420 - 3 847 846)	1 677 406 (1 590 104 - 1 763 072)	830 331 (673 665 - 1 017 454)	426 495 (337 669 - 526 923)	144 707 (133 447 - 153 606)	14 243 (12 856 - 15 855)
Aortic aneurysm	159 692 (139 029 - 183 282)	42 790 (6 987 - 58 396)	23 614 (19 345 - 28 070)	18 674 (13 736 - 25 838)	8 902 (6 674 - 11 889)	39 423 (30 282 - 50 091)	20 065 (16 959 - 23 618)	1 719 (1 101 - 2 730)	836 (590 - 1 194)	3 344 (3 054 - 3 584)	324 (277 - 386)
Atrial fibrillation and flutter	591 249 (483 702 - 718 597)	223 354 (178 284 - 274 232)	72 144 (56 995 - 88 259)	97 149 (74 777 - 122 074)	43 946 (34 393 - 56 178)	110 567 (87 502 - 136 071)	26 502 (20 978 - 32 395)	9 138 (7 031 - 11 349)	3 842 (2 968 - 4 839)	4 278 (3 235 - 5 584)	328 (271 - 388)
Cardiomyopathy and myocarditis	505 076 (431 799 - 593 178)	215 019 (164 992 - 283 257)	93 229 (71 325 - 108 233)	79 453 (59 467 - 103 090)	49 854 (37 827 - 67 561)	23 924 (17 089 - 30 966)	24 182 (20 670 - 28 165)	9 473 (6 735 - 13 394)	5 248 (3 742 - 7 538)	3 895 (3 542 - 4 246)	799 (687 - 946)
Endocarditis	246 276 (195 776 - 351 258)	77 190 (48 486 - 135 622)	34 095 (22 994 - 41 928)	25 476 (14 696 - 46 900)	20 383 (12 458 - 35 591)	65 797 (48 830 - 90 728)	15 002 (11 374 - 18 361)	4 387 (2 604 - 7 812)	2 971 (1 623 - 5 130)	848 (774 - 911)	127 (101 - 173)
Hypertensive heart disease	2 873 308 (2 035 972 - 3 366 557)	1 406 681 (886 661 - 1 835 952)	597 858 (494 705 - 708 972)	377 413 (243 730 - 557 095)	270 308 (141 519 - 395 142)	72 758 (56 726 - 91 612)	32 784 (27 717 - 38 925)	73 435 (45 646 - 99 542)	30 719 (19 174 - 41 815)	10 729 (9 654 - 11 605)	623 (508 - 808)
Ischemic heart disease	15 407 244 (13 831 551 - 16 926 728)	7 347 522 (6 101 893 - 8 695 820)	2 841 769 (2 400 536 - 3 317 750)	1 388 815 (1 115 926 - 1 666 230)	1 313 712 (1 045 166 - 1 653 587)	1 082 564 (855 053 - 1 334 623)	901 888 (844 940 - 952 014)	271 596 (210 702 - 344 178)	173 831 (133 708 - 222 410)	79 251 (73 676 - 83 494)	6 297 (5 562 - 7 068)
Lower extremity peripheral arterial disease	56 789 (33 819 - 95 755)	22 142 (13 204 - 37 168)	8 635 (5 679 - 13 901)	8 835 (5 414 - 14 975)	4 543 (2 798 - 7 853)	7 535 (3 955 - 13 757)	2 501 (1 489 - 4 280)	1 012 (543 - 1 799)	425 (250 - 732)	1 127 (920 - 1 457)	36 (26 - 51)
Non-rheumatic valvular heart disease	48 683 (38 367 - 74 494)	12 492 (8 054 - 28 099)	4 808 (3 729 - 5 877)	4 748 (3 074 - 10 400)	2 682 (1 799 - 5 960)	17 559 (12 862 - 23 175)	3 957 (3 399 - 4 739)	534 (336 - 1 217)	256 (160 - 620)	1 468 (1 244 - 1 792)	179 (139 - 214)
Other cardiovascular and circulatory diseases	384 876 (318 077 - 498 354)	146 277 (113 296 - 212 865)	53 013 (44 461 - 62 692)	49 511 (36 356 - 69 792)	36 956 (27 259 - 57 219)	52 056 (40 508 - 68 352)	32 815 (27 438 - 39 387)	6 988 (5 251 - 10 141)	3 983 (2 741 - 6 212)	2 981 (2 207 - 4 008)	297 (246 - 365)
Pulmonary Arterial Hypertension	27 275 (19 753 - 55 345)	11 947 (7 868 - 24 908)	3 531 (2 584 - 7 427)	3 747 (2 123 - 8 856)	3 636 (2 377 - 7 104)	2 149 (1 425 - 6 094)	757 (519 - 1 871)	777 (500 - 1 513)	479 (256 - 980)	207 (187 - 228)	45 (27 - 58)
Rheumatic heart disease	467 302 (376 029 - 581 963)	179 418 (136 556 - 249 802)	90 016 (64 926 - 120 686)	38 773 (28 468 - 51 352)	72 180 (54 918 - 95 641)	35 164 (25 826 - 48 431)	23 514 (18 202 - 30 475)	16 099 (11 533 - 21 733)	11 063 (7 769 - 16 536)	919 (819 - 1 015)	157 (132 - 186)

Stroke	21 657 336 (19 386 706 - 23 716 083)	10 624 062 (8 956 510 - 12 309 257)	2 364 482 (2 036 639 - 2 715 267)	3 768 751 (3 131 538 - 4 444 248)	1 961 453 (1 593 525 - 2 424 347)	1 676 448 (1 346 657 - 2 042 439)	593 439 (541 123 - 650 113)	435 172 (339 040 - 534 642)	192 841 (153 953 - 240 429)	35 659 (31 474 - 39 597)	5 029 (4 390 - 5 832)

Table S14 Age-standardised DALY rates (per 100 000 population) of CVD causes in 2021

	Association of Southeast Asian Nations	Laos	Indonesia	Myanmar	Philippines	Cambodia	Viet Nam	Malaysia	Brunei	Thailand	Singapore
Cardiovascular diseases	6 735.6 (6 111.4 - 7 291.6)	8 921.9 (7 189.7 - 10 805.3)	8 680.4 (7 378.5 - 9 908.1)	7 873.6 (6 574.2 - 9 553.1)	7 252.9 (6 231.6 - 8 292.0)	6 971.0 (5 737.5 - 8 341.6)	6 181.9 (5 223.1 - 7 097.2)	5 946.1 (5 641.6 - 6 246.6)	4 115.5 (3 735.9 - 4 557.4)	3 126.6 (2 552.9 - 3 754.9)	1 730.9 (1 593.0 - 1 839.6)
Aortic aneurysm	27.0 (23.7 - 31.1)	18.2 (13.2 - 25.3)	19.4 (13.3 - 26.2)	19.0 (14.5 - 25.1)	28.4 (23.2 - 33.5)	14.5 (9.2 - 23.2)	20.1 (14.8 - 27.6)	74.1 (62.2 - 87.8)	93.4 (78.8 - 110.2)	38.8 (30.1 - 49.3)	39.8 (36.2 - 42.6)
Atrial fibrillation and flutter	115.2 (95.9 - 138.9)	111.2 (87.6 - 138.4)	133.1 (106.8 - 160.6)	111.5 (88.0 - 140.6)	111.0 (89.2 - 135.2)	98.5 (76.5 - 122.3)	118.9 (91.5 - 147.6)	112.6 (91.2 - 135.6)	122.0 (102.5 - 144.0)	101.0 (80.1 - 124.4)	50.5 (38.4 - 65.6)
Cardiomyopathy and myocarditis	81.4 (70.6 - 94.3)	96.4 (70.2 - 133.8)	95.7 (74.7 - 121.4)	102.7 (79.5 - 136.5)	103.8 (76.4 - 120.3)	74.5 (53.0 - 103.5)	85.1 (64.4 - 107.5)	83.7 (71.5 - 96.7)	197.5 (169.9 - 232.7)	28.8 (19.8 - 37.0)	55.9 (51.2 - 61.1)
Endocarditis	36.9 (29.3 - 52.7)	42.9 (23.4 - 74.6)	28.1 (17.4 - 50.0)	37.2 (22.7 - 64.9)	32.1 (22.1 - 39.0)	28.7 (16.7 - 52.2)	24.7 (14.3 - 45.8)	47.1 (35.7 - 57.0)	34.1 (27.9 - 44.0)	77.8 (59.1 - 105.6)	11.0 (10.0 - 11.8)
Hypertensive heart disease	466.5 (335.4 - 541.1)	690.3 (427.3 - 933.1)	632.3 (405.4 - 817.6)	575.3 (305.5 - 831.1)	738.9 (618.4 - 869.2)	631.4 (392.2 - 834.5)	410.4 (269.0 - 590.4)	118.9 (101.2 - 140.7)	223.3 (182.3 - 284.7)	68.3 (53.3 - 86.0)	126.1 (113.4 - 136.6)
Ischemic heart disease	2 413.3 (2 177.8 - 2 647.5)	3 667.1 (2 896.8 - 4 573.2)	3 043.1 (2 527.3 - 3 544.7)	2 773.1 (2 241.1 - 3 446.1)	3 326.5 (2 831.6 - 3 850.5)	2 251.2 (1 767.8 - 2 790.1)	1 464.6 (1 198.7 - 1 740.5)	3 180.4 (2 984.1 - 3 352.2)	1 726.3 (1 525.6 - 1 929.9)	1 039.7 (825.6 - 1 278.9)	934.1 (866.5 - 985.5)
Lower extremity peripheral arterial disease	9.9 (5.8 - 16.9)	10.7 (6.2 - 18.5)	10.9 (6.4 - 18.7)	10.3 (6.2 - 17.7)	11.8 (7.5 - 19.4)	9.3 (5.0 - 16.9)	9.7 (5.8 - 16.4)	9.6 (5.7 - 16.4)	13.9 (10.3 - 19.3)	6.8 (3.6 - 12.4)	13.5 (11.0 - 17.3)
Non-rheumatic valvular heart disease	8.5 (6.7 - 12.9)	5.7 (3.6 - 13.0)	6.1 (3.9 - 13.5)	5.9 (4.0 - 12.9)	6.0 (4.6 - 7.5)	4.8 (3.1 - 10.8)	5.4 (3.5 - 11.7)	14.4 (12.3 - 17.4)	65.8 (49.3 - 78.0)	17.6 (13.0 - 23.2)	17.9 (15.2 - 21.9)
Other cardiovascular and circulatory diseases	59.5 (49.4 - 77.6)	63.2 (44.7 - 99.3)	57.6 (44.6 - 84.7)	70.1 (51.8 - 108.3)	51.9 (44.1 - 61.5)	48.8 (36.7 - 70.6)	49.4 (36.4 - 70.7)	110.6 (92.5 - 132.6)	74.5 (62.3 - 89.3)	58.0 (45.5 - 74.1)	38.3 (28.8 - 50.9)
Pulmonary Arterial Hypertension	4.2 (3.0 - 9.1)	6.7 (3.7 - 14.0)	4.6 (3.0 - 10.4)	6.8 (4.4 - 13.8)	3.3 (2.4 - 7.5)	4.9 (3.1 - 10.1)	3.8 (2.1 - 9.6)	2.5 (1.7 - 6.6)	11.4 (7.2 - 14.9)	3.1 (2.1 - 7.5)	3.8 (3.4 - 4.3)
Rheumatic heart disease	66.7 (53.8 - 82.9)	147.9 (104.0 - 220.2)	62.5 (47.4 - 86.3)	125.3 (95.5 - 165.6)	77.5 (55.7 - 104.4)	94.9 (68.0 - 127.0)	37.4 (27.7 - 48.9)	69.9 (54.3 - 90.0)	44.1 (37.6 - 51.4)	50.6 (37.2 - 70.2)	11.5 (10.3 - 12.7)

Stroke	3 446.4 (3 091.1 - 3 761.3)	4 061.5 (3 276.8 - 4 988.7)	4 587.1 (3 889.4 - 5 248.2)	4 036.4 (3 276.2 - 4 964.3)	2 761.8 (2 384.5 - 3 156.6)	3 709.4 (2 911.2 - 4 460.1)	3 952.5 (3 287.4 - 4 604.9)	2 122.3 (1 934.6 - 2 323.9)	1 509.0 (1 324.4 - 1 727.1)	1 636.0 (1 316.7 - 1 984.0)	428.6 (377.9 - 476.4)

Table S15: Percentage change of age-standardised DALY rates (per 100 000 population) of CVD causes from 1990 to 2021

	Association of Southeast Asian Nations	Brunei	Cambodia	Thailand	Laos	Malaysia	Indonesia	Singapore	Philippines	Myanmar	Viet Nam
Cardiovascular diseases	-17.0 (-26.1 to -6.9)	-45.8 (-52.4 to -36.9)	-25.4 (-40.6 to -6.0)	-37.0 (-49.4 to -22.1)	-39.1 (-53.2 to -19.2)	-26.1 (-29.7 to -21.4)	2.6 (-14.5 to 22.4)	-71.9 (-73.3 to -70.6)	-5.1 (-18.9 to 10.8)	-40.3 (-54.0 to -22.0)	-13.4 (-32.7 to 9.9)
Aortic aneurysm	31.8 (2.0 to 67.0)	-13.0 (-36.0 to 22.0)	28.7 (-13.9 to 102.6)	8.9 (-25.5 to 62.3)	12.7 (-26.0 to 79.7)	14.8 (-16.2 to 54.4)	50.3 (-3.7 to 118.2)	-28.0 (-34.1 to -22.2)	18.9 (-5.3 to 47.8)	15.3 (-23.3 to 87.6)	58.1 (7.0 to 146.4)
Atrial fibrillation and flutter	12.3 (-1.8 to 26.4)	-15.6 (-28.9 to 2.6)	14.1 (-3.3 to 33.0)	-10.4 (-28.9 to 8.0)	8.6 (-10.0 to 32.3)	22.0 (9.5 to 39.4)	31.0 (11.4 to 52.8)	-26.8 (-32.0 to -22.4)	-0.7 (-10.7 to 8.9)	8.1 (-13.6 to 30.6)	23.9 (4.8 to 47.8)
Cardiomyopathy and myocarditis	-9.6 (-27.4 to 17.9)	-35.9 (-48.7 to -20.1)	-9.0 (-36.1 to 50.1)	45.5 (-21.3 to 126.8)	-17.8 (-47.1 to 39.3)	-38.2 (-52.2 to -17.6)	10.8 (-20.7 to 57.2)	-77.6 (-79.6 to -75.4)	-21.9 (-34.8 to -5.1)	-17.1 (-41.3 to 26.5)	-5.5 (-36.7 to 44.4)
Endocarditis	-25.3 (-34.9 to -14.4)	-21.1 (-42.2 to 5.4)	-23.0 (-47.1 to 2.8)	-31.1 (-47.9 to -9.3)	-16.6 (-43.9 to 18.5)	-26.9 (-38.5 to -10.7)	-21.1 (-37.5 to -3.3)	-14.1 (-21.4 to -7.1)	-6.6 (-21.6 to 10.9)	-33.4 (-54.1 to -4.2)	-31.1 (-51.8 to -4.9)
Hypertensive heart disease	-23.1 (-35.7 to 0.5)	-39.5 (-54.7 to -14.9)	-30.4 (-51.5 to 5.0)	-28.0 (-47.1 to 7.4)	-42.9 (-61.7 to 22.5)	-38.2 (-51.3 to -11.5)	-7.4 (-27.7 to 26.2)	-57.7 (-61.5 to -54.4)	1.2 (-15.9 to 21.0)	-42.4 (-58.8 to -4.6)	-33.5 (-53.9 to -2.0)
Ischemic heart disease	-4.7 (-16.8 to 8.0)	-44.7 (-53.3 to -34.3)	-13.7 (-34.8 to 11.1)	-38.3 (-52.3 to -21.0)	-32.9 (-50.2 to -7.4)	-18.0 (-25.0 to -10.7)	25.7 (0.8 to 54.8)	-70.6 (-72.1 to -69.1)	-5.5 (-19.8 to 12.4)	-36.5 (-52.8 to -12.7)	10.3 (-16.3 to 44.0)
Lower extremity peripheral arterial disease	6.3 (-1.9 to 20.9)	-25.7 (-40.5 to 1.5)	7.0 (-6.4 to 24.9)	-8.0 (-15.1 to 2.1)	7.5 (-4.5 to 29.3)	5.6 (-4.5 to 21.2)	17.7 (4.0 to 41.7)	-28.0 (-33.8 to -21.5)	7.2 (-1.4 to 23.5)	-10.2 (-21.9 to 5.2)	15.5 (0.3 to 39.3)
Non-rheumatic valvular heart disease	18.2 (-6.2 to 51.0)	-5.7 (-23.5 to 18.9)	-9.2 (-30.8 to 17.6)	75.3 (10.3 to 171.8)	-16.5 (-40.3 to 17.1)	-10.8 (-27.2 to 16.0)	4.2 (-16.3 to 29.8)	-56.9 (-60.8 to -52.5)	19.0 (2.7 to 36.7)	-15.7 (-39.2 to 13.8)	2.2 (-19.5 to 31.6)
Other cardiovascular and circulatory diseases	-6.9 (-18.3 to 9.0)	-10.6 (-25.6 to 8.4)	-15.0 (-34.1 to 17.7)	1.4 (-14.7 to 20.9)	-26.2 (-45.9 to 13.3)	4.3 (-11.6 to 19.6)	-9.1 (-25.3 to 10.6)	-37.0 (-47.7 to -23.9)	-5.7 (-17.9 to 8.5)	-21.5 (-40.7 to 12.2)	-2.9 (-23.3 to 17.6)
Pulmonary Arterial Hypertension	-28.4 (-43.0 to -5.0)	-44.5 (-58.8 to -17.7)	-26.1 (-54.2 to 22.1)	-25.4 (-46.7 to 7.0)	-33.2 (-55.9 to 13.8)	-23.1 (-46.5 to 4.0)	-23.0 (-42.8 to 10.4)	-67.1 (-71.2 to -62.7)	-33.6 (-47.0 to -14.3)	-33.3 (-54.0 to 9.4)	-29.5 (-53.9 to 9.9)
Rheumatic heart disease	-58.5 (-66.7 to -46.0)	-51.5 (-64.6 to -34.2)	-62.0 (-73.6 to -44.8)	-31.4 (-49.1 to -10.3)	-62.5 (-74.6 to -43.5)	-55.2 (-64.4 to -46.1)	-62.6 (-71.3 to -48.2)	-79.5 (-81.7 to -77.5)	-40.8 (-51.2 to -28.7)	-66.5 (-76.2 to -51.5)	-63.5 (-75.4 to -45.6)
Stroke	-22.8 (-31.4 to -12.9)	-53.0 (-60.1 to -43.3)	-29.8 (-45.5 to -11.9)	-40.5 (-52.8 to -24.2)	-43.3 (-57.6 to -26.5)	-36.1 (-42.2 to -28.7)	-5.8 (-20.9 to 12.4)	-79.6 (-81.3 to -78.0)	-4.2 (-17.1 to 11.3)	-42.6 (-56.5 to -23.5)	-17.4 (-36.5 to 5.5)

Table S16: Percentage change of the number of DALYs attributed to CVD causes from 1990 to 2021

	Association of Southeast Asian Nations	Brunei	Cambodia	Thailand	Laos	Malaysia	Indonesia	Singapore	Philippines	Myanmar	Viet Nam
Cardiovascular diseases	100.7 (78.3 to 122.8)	60.9 (39.5 to 88.7)	83.4 (44.5 to 132.7)	76.4 (41.8 to 120.7)	27.3 (-3.6 to 72.3)	113.7 (102.5 to 127.4)	124.7 (88.2 to 167.5)	4.9 (-0.9 to 9.7)	155.6 (117.0 to 199.2)	14.9 (-12.4 to 51.4)	105.3 (58.2 to 161.6)
Aortic aneurysm	224.1 (145.2 to 315.0)	178.3 (98.7 to 310.0)	239.9 (118.5 to 442.4)	225.0 (120.3 to 382.4)	151.7 (60.7 to 326.9)	239.7 (147.2 to 354.4)	235.4 (111.1 to 399.7)	170.4 (145.9 to 193.2)	220.9 (151.1 to 300.4)	131.4 (53.3 to 288.2)	268.0 (146.4 to 474.3)
Atrial fibrillation and flutter	206.2 (171.5 to 240.2)	163.9 (122.4 to 218.1)	212.2 (165.4 to 261.4)	266.7 (197.8 to 342.9)	150.1 (111.2 to 197.7)	252.7 (219.2 to 298.1)	196.5 (157.9 to 236.3)	209.4 (187.3 to 229.0)	212.0 (181.9 to 247.0)	144.6 (101.3 to 193.3)	193.9 (150.8 to 248.8)
Cardiomyopathy and myocarditis	88.3 (42.3 to 147.3)	36.4 (8.2 to 71.2)	91.1 (13.0 to 228.0)	165.6 (55.2 to 323.1)	64.3 (-12.4 to 199.9)	43.9 (12.5 to 90.4)	104.6 (31.3 to 195.5)	-43.5 (-48.7 to -37.8)	97.6 (63.9 to 137.0)	44.9 (-8.7 to 127.5)	98.0 (33.3 to 203.7)
Endocarditis	39.4 (18.9 to 63.2)	95.1 (39.1 to 167.0)	62.0 (8.2 to 125.9)	21.7 (-10.5 to 62.6)	79.9 (17.0 to 165.1)	63.5 (34.8 to 103.3)	44.9 (11.9 to 85.6)	169.1 (145.0 to 191.6)	95.0 (62.7 to 129.3)	5.9 (-29.5 to 55.8)	37.0 (-6.5 to 88.5)
Hypertensive heart disease	98.7 (65.2 to 161.3)	73.4 (27.2 to 158.2)	84.6 (27.1 to 188.9)	128.7 (66.8 to 244.6)	25.1 (-18.3 to 185.2)	88.1 (46.8 to 165.2)	115.7 (66.3 to 194.2)	70.1 (54.1 to 84.5)	186.5 (136.2 to 245.5)	18.5 (-16.4 to 102.7)	59.2 (10.2 to 134.7)
Ischemic heart disease	135.5 (105.2 to 167.6)	72.6 (43.7 to 108.1)	121.0 (65.8 to 193.6)	82.0 (40.5 to 133.2)	46.0 (6.3 to 107.7)	147.5 (126.7 to 171.6)	182.4 (123.6 to 249.4)	10.8 (4.4 to 16.6)	160.6 (119.2 to 213.2)	24.8 (-8.7 to 74.1)	166.0 (99.2 to 254.5)
Lower extremity peripheral arterial disease	190.9 (166.4 to 236.4)	136.5 (88.5 to 209.7)	208.0 (166.5 to 265.0)	238.5 (208.6 to 278.7)	141.7 (109.3 to 198.4)	239.0 (207.7 to 285.0)	198.4 (160.2 to 268.7)	223.1 (195.1 to 252.3)	207.9 (181.9 to 260.7)	98.0 (70.7 to 136.1)	178.5 (141.2 to 240.9)
Non-rheumatic valvular heart disease	175.3 (115.7 to 260.0)	153.9 (102.9 to 224.0)	122.9 (60.4 to 216.5)	361.8 (191.8 to 629.8)	80.2 (20.1 to 175.3)	144.2 (98.5 to 217.3)	118.5 (66.5 to 194.5)	69.6 (53.0 to 88.7)	225.2 (173.8 to 281.9)	59.2 (8.6 to 133.7)	130.2 (77.7 to 204.0)
Other cardiovascular and circulatory diseases	65.5 (38.1 to 104.3)	117.0 (79.3 to 166.7)	51.1 (6.5 to 138.5)	99.2 (63.5 to 146.9)	24.5 (-17.1 to 130.4)	142.1 (106.9 to 183.8)	55.7 (22.2 to 102.4)	94.5 (58.9 to 136.3)	67.6 (39.0 to 103.5)	16.7 (-17.0 to 86.0)	88.9 (50.1 to 133.6)
Pulmonary Arterial Hypertension	8.4 (-20.7 to 52.8)	-15.6 (-38.4 to 30.6)	7.5 (-41.6 to 97.2)	2.1 (-31.0 to 50.3)	0.1 (-39.4 to 97.4)	48.3 (-0.7 to 103.7)	15.5 (-20.5 to 73.1)	-33.4 (-40.5 to -25.4)	9.4 (-14.1 to 44.1)	-12.1 (-43.9 to 56.4)	14.7 (-26.6 to 88.0)
Rheumatic heart disease	-32.4 (-45.7 to -12.2)	26.0 (-8.9 to 74.4)	-28.4 (-49.6 to 1.7)	-20.0 (-39.3 to 4.0)	-27.2 (-51.1 to 10.1)	-6.7 (-25.2 to 12.0)	-38.8 (-53.8 to -15.0)	-32.7 (-40.0 to -25.5)	11.7 (-8.1 to 36.9)	-52.0 (-65.6 to -29.5)	-33.8 (-55.1 to -2.8)
Stroke	88.7 (67.2 to 110.9)	41.6 (19.1 to 72.6)	73.6 (33.0 to 121.3)	68.3 (33.4 to 113.6)	16.5 (-13.3 to 54.8)	84.4 (66.6 to 107.0)	107.2 (74.8 to 145.4)	-22.2 (-28.4 to -16.4)	160.7 (122.7 to 203.7)	12.1 (-15.6 to 50.6)	97.8 (51.0 to 154.8)

Table S17: Age-standardised mortality rates (per 100 000 population) attributed to cardiovascular risk factors in 2021

	Association of Southeast Asian Nations	Laos	Indonesia	Myanmar	Cambodia	Philippines	Viet Nam	Malaysia	Brunei	Thailand	Singapore
Air pollution	72.8 (54.5 - 91.0)	151.8 (106.4 - 194.5)	83.1 (57.5 - 112.1)	136.7 (102.3 - 176.0)	130.4 (100.0 - 158.0)	84.5 (61.5 - 106.0)	79.5 (56.6 - 101.3)	38.6 (26.2 - 53.3)	7.7 (1.6 - 15.2)	28.8 (20.6 - 38.9)	9.6 (5.2 - 14.2)
Dietary risks	80.4 (18.8 - 127.0)	117.9 (20.1 - 190.7)	109.1 (19.8 - 176.2)	100.2 (27.1 - 161.7)	108.9 (36.0 - 168.6)	98.7 (26.2 - 155.6)	67.6 (25.8 - 109.8)	72.5 (8.7 - 118.9)	56.8 (15.6 - 88.9)	26.9 (4.2 - 47.5)	22.4 (6.0 - 34.5)
High alcohol use	4.9 (1.1 - 9.2)	10.6 (0.9 - 22.2)	0.6 (0.0 - 1.5)	6.2 (1.5 - 11.9)	9.2 (2.8 - 16.7)	7.4 (1.2 - 15.1)	16.0 (3.9 - 28.7)	0.5 (-0.5 - 1.7)	0.6 (0.2 - 1.1)	2.6 (0.4 - 5.3)	-0.2 (-0.7 - 0.3)
High body-mass index	15.9 (10.1 - 23.3)	21.7 (12.7 - 34.0)	19.6 (12.1 - 28.6)	15.8 (9.5 - 24.5)	12.7 (8.0 - 18.8)	27.3 (17.7 - 39.4)	8.0 (4.7 - 12.0)	20.8 (8.6 - 33.5)	14.5 (7.7 - 21.7)	7.4 (3.0 - 12.9)	7.6 (4.6 - 11.1)
High fasting plasma glucose	29.8 (25.0 - 34.5)	43.3 (34.5 - 53.2)	39.9 (32.0 - 48.1)	42.1 (33.6 - 53.6)	28.4 (22.4 - 35.0)	27.6 (23.4 - 33.0)	27.7 (21.4 - 33.7)	43.1 (37.5 - 49.3)	29.3 (24.5 - 34.7)	13.1 (10.0 - 16.5)	10.8 (9.2 - 12.4)
High LDL cholesterol	48.1 (26.4 - 71.1)	70.0 (38.7 - 107.9)	54.5 (28.2 - 82.5)	57.2 (30.2 - 88.9)	48.7 (24.8 - 77.3)	62.4 (37.9 - 90.1)	43.9 (20.1 - 71.5)	69.9 (43.3 - 97.4)	39.0 (21.7 - 57.4)	24.7 (13.3 - 38.0)	17.7 (11.3 - 24.5)
High systolic blood pressure	184.8 (153.5 - 212.1)	231.7 (172.2 - 285.2)	276.8 (222.9 - 327.3)	212.7 (169.4 - 266.6)	177.4 (135.5 - 219.4)	163.3 (129.6 - 196.8)	195.8 (154.1 - 233.9)	166.5 (139.7 - 188.5)	96.4 (77.3 - 116.3)	59.3 (42.8 - 77.8)	31.3 (24.3 - 38.5)
Kidney dysfunction	38.9 (30.5 - 48.1)	56.6 (43.4 - 73.4)	54.1 (41.2 - 68.3)	47.6 (35.8 - 63.1)	41.1 (30.6 - 51.3)	42.9 (33.7 - 53.2)	31.1 (22.7 - 40.4)	42.6 (33.3 - 51.6)	22.0 (16.6 - 27.6)	18.0 (13.3 - 23.7)	9.5 (6.7 - 12.0)
Low physical activity	6.8 (2.2 - 11.9)	2.5 (0.5 - 5.8)	16.2 (5.5 - 28.5)	1.6 (0.3 - 3.5)	1.8 (-0.2 - 4.5)	2.6 (0.7 - 5.2)	2.8 (0.1 - 6.3)	5.0 (1.9 - 9.3)	3.1 (0.6 - 6.5)	1.9 (0.4 - 4.0)	0.8 (0.3 - 1.6)
Non-optimal temperature	5.3 (4.1 - 6.9)	12.6 (9.2 - 16.9)	3.4 (2.8 - 4.4)	12.2 (8.7 - 16.8)	9.7 (5.6 - 12.7)	3.1 (2.6 - 3.7)	10.5 (5.7 - 16.4)	1.8 (1.6 - 2.3)	0.6 (0.4 - 0.9)	3.6 (2.3 - 5.2)	0.4 (0.2 - 0.5)
Other environmental risks	19.7 (-2.4 - 41.9)	38.9 (-4.2 - 79.2)	29.7 (-3.9 - 63.9)	29.8 (-3.2 - 61.7)	31.5 (-3.0 - 64.0)	17.7 (-1.6 - 36.0)	18.8 (-2.2 - 40.2)	12.9 (-1.8 - 27.6)	9.7 (-1.0 - 20.8)	4.5 (-0.5 - 10.6)	5.6 (-0.5 - 11.5)
Tobacco	47.6 (38.7 - 57.1)	71.7 (52.3 - 92.7)	64.4 (48.5 - 80.8)	46.0 (33.0 - 60.5)	59.7 (44.0 - 74.7)	49.7 (39.1 - 61.3)	49.9 (37.7 - 61.9)	40.5 (32.7 - 49.8)	22.5 (17.4 - 28.8)	18.5 (13.5 - 25.2)	7.0 (5.7 - 8.7)

Table S18: Age-standardised DALY rates (per 100 000 population) attributed to cardiovascular risk factors in 2021

	Association of Southeast Asian Nations	Laos	Indonesia	Myanmar	Philippines	Cambodia	Viet Nam	Malaysia	Brunei	Thailand	Singapore
Air pollution	1 572.2 (1 181.0 - 1 988.3)	3 195.1 (2 191.0 - 4 157.2)	1 731.4 (1 193.4 - 2 344.7)	2 860.4 (2 126.4 - 3 678.8)	1 892.2 (1 382.4 - 2 396.4)	2 591.7 (1 975.3 - 3 201.3)	1 554.7 (1 105.3 - 2 004.7)	830.5 (559.7 - 1 144.2)	158.0 (33.3 - 311.4)	667.8 (481.5 - 895.8)	206.6 (110.2 - 306.4)
Dietary risks	1 882.5 (389.3 - 2 933.1)	2 585.6 (387.8 - 4 205.8)	2 488.6 (400.0 - 4 045.9)	2 227.6 (571.8 - 3 524.0)	2 313.0 (465.2 - 3 604.2)	2 364.5 (671.6 - 3 681.5)	1 383.2 (562.3 - 2 216.0)	1 694.5 (184.7 - 2 719.5)	1 224.1 (247.6 - 1 892.1)	664.9 (108.0 - 1 137.1)	494.5 (132.1 - 743.1)
High alcohol use	119.2 (21.6 - 228.7)	258.4 (2.2 - 557.5)	14.7 (-0.7 - 37.5)	161.6 (35.7 - 318.2)	189.8 (7.1 - 392.3)	216.1 (58.1 - 398.5)	373.5 (80.2 - 688.4)	12.0 (-14.6 - 46.5)	19.5 (5.8 - 33.9)	76.8 (7.0 - 155.8)	-3.8 (-20.4 - 13.1)
High body-mass index	436.1 (257.9 - 646.7)	564.9 (319.9 - 893.4)	527.1 (310.0 - 775.6)	421.1 (244.6 - 651.3)	734.3 (442.1 - 1 080.8)	322.0 (203.7 - 463.9)	186.8 (112.8 - 281.5)	546.4 (219.2 - 875.1)	414.8 (194.6 - 631.1)	221.4 (81.1 - 391.1)	197.1 (114.5 - 288.5)
High fasting plasma glucose	560.0 (473.5 - 655.3)	782.3 (620.8 - 966.2)	727.7 (586.3 - 881.0)	773.7 (614.5 - 985.4)	520.7 (437.1 - 627.4)	481.6 (381.4 - 603.5)	467.5 (360.6 - 572.7)	830.6 (723.3 - 944.5)	537.9 (453.2 - 632.2)	259.9 (200.4 - 319.5)	219.0 (189.0 - 248.5)
High LDL cholesterol	1 183.6 (709.3 - 1 654.1)	1 669.3 (1 006.8 - 2 455.7)	1 327.2 (767.8 - 1 922.1)	1 308.0 (756.4 - 1 959.9)	1 595.8 (1 044.9 - 2 221.5)	1 078.1 (591.5 - 1 651.2)	922.1 (472.2 - 1 433.0)	1 706.0 (1 149.3 - 2 246.5)	922.3 (585.3 - 1 279.3)	645.3 (388.2 - 923.9)	440.4 (292.3 - 581.8)
High systolic blood pressure	3 914.3 (3 239.4 - 4 534.2)	4 775.8 (3 470.9 - 5 951.2)	5 589.7 (4 488.1 - 6 681.5)	4 368.3 (3 416.5 - 5 531.8)	3 563.7 (2 841.6 - 4 347.1)	3 293.7 (2 437.8 - 4 127.7)	3 787.3 (2 973.8 - 4 585.1)	3 564.7 (3 000.9 - 4 030.1)	1 923.3 (1 523.7 - 2 310.2)	1 332.0 (980.7 - 1 751.5)	653.6 (519.3 - 793.4)
Kidney dysfunction	823.5 (650.8 - 1 005.1)	1 148.5 (864.7 - 1 504.4)	1 100.3 (846.7 - 1 399.6)	956.1 (724.6 - 1 281.5)	925.3 (728.1 - 1 135.9)	794.3 (590.6 - 1 003.1)	611.8 (446.2 - 792.3)	868.3 (697.2 - 1 052.9)	408.0 (314.7 - 499.2)	386.5 (289.4 - 500.3)	182.5 (133.9 - 225.1)
Low physical activity	139.1 (55.8 - 231.8)	49.6 (17.2 - 98.8)	305.4 (121.7 - 518.2)	32.7 (10.6 - 63.2)	53.8 (21.1 - 94.5)	30.6 (4.2 - 68.2)	59.8 (17.2 - 115.1)	113.1 (48.2 - 187.4)	61.6 (20.7 - 115.8)	37.7 (13.6 - 71.4)	21.4 (9.1 - 36.8)
Non-optimal temperature	105.8 (83.8 - 138.0)	259.1 (185.7 - 354.7)	69.5 (55.9 - 89.6)	249.8 (174.9 - 346.6)	68.8 (56.2 - 81.6)	186.2 (106.8 - 250.0)	196.6 (104.8 - 307.5)	36.9 (31.8 - 46.6)	10.1 (5.9 - 14.7)	78.2 (49.1 - 110.3)	7.7 (3.8 - 9.3)
Other environmental risks	416.8 (-49.6 - 878.5)	786.4 (-85.0 - 1 600.9)	594.1 (-77.6 - 1 278.0)	610.6 (-65.5 - 1 268.9)	383.5 (-34.2 - 782.8)	617.8 (-58.1 - 1 266.6)	366.8 (-41.6 - 772.5)	270.4 (-37.9 - 588.8)	167.5 (-18.2 - 353.2)	99.8 (-11.7 - 230.6)	107.7 (-10.9 - 219.6)

Tobacco	1 273.0 (1 037.5 - 1 508.3)	1 787.6 (1 324.0 - 2 327.3)	1 642.2 (1 265.1 - 2 068.6)	1 142.0 (830.7 - 1 492.6)	1 405.5 (1 108.8 - 1 721.0)	1 399.4 (1 042.9 - 1 790.2)	1 239.4 (943.5 - 1 543.7)	1 078.8 (891.1 - 1 293.5)	640.7 (508.2 - 808.7)	550.5 (406.4 - 728.9)	210.2 (173.0 - 253.7)

Table S19: Percentage change of age-standardised DALY rates (per 100 000 population) of cardiovascular risk factors from 1990 to 2021

	Association of Southeast Asian Nations	Brunei	Cambodia	Indonesia	Laos	Malaysia	Myanmar	Philippines	Singapore	Thailand	Viet Nam
Air pollution	-47.4 (-57.8 to -35.3)	-52.7 (-90.5 to 366.7)	-31.5 (-46.3 to - 15.0)	-42.5 (-58.8 to - 22.4)	-46.3 (-62.8 to -27.6)	-53.9 (-73.5 to - 4.1)	-46.5 (-60.3 to - 28.4)	-28.9 (-46.1 to - 11.5)	-85.3 (-93.2 to - 59.9)	-63.7 (-73.4 to - 51.1)	-47.3 (-62.6 to - 29.5)
Alcohol use	165.4 (-674.0 to 1148.9)	-65.4 (-82.3 to - 33.3)	252.2 (-685.1 to 1081.7)	-13.1 (-83.0 to 50.9)	11.2 (-300.7 to 286.2)	-75.3 (-234.5 to 65.0)	520.2 (-3488.8 to 3181.3)	52.7 (-300.5 to 504.6)	-206.5 (-302.1 to 138.3)	-19.7 (-48.7 to 42.7)	2787.1 (-8022.7 to 23887.4)
Dietary risks	-34.5 (-53.9 to -22.2)	-43.8 (-55.3 to - 31.7)	-35.9 (-53.6 to - 16.9)	-18.5 (-50.1 to 4.9)	-58.5 (-77.3 to -41.5)	-39.8 (-66.0 to - 32.0)	-56.4 (-70.4 to - 38.9)	-16.3 (-31.8 to - 1.1)	-73.8 (-76.1 to - 69.9)	-52.8 (-70.0 to - 36.6)	-40.2 (-58.6 -19.6)
High body-mass index	64.0 (24.5 - 103.4)	7.7 (-16.7 to 33.2)	15.9 (-22.0 to 74.2)	112.4 (46.4 to 199.5)	16.6 (-24.9 to 90.7)	20.1 (1.5 to 38.1)	-11.2 (-39.7 to 33.8)	64.0 (31.9 to 100.0)	-34.7 (-43.4 to - 26.2)	63.4 (14.5 to 125.0)	81.9 (4.0 - 190.6)
High fasting plasma glucose	15.1 (2.0 - 31.3)	-39.4 (-48.6 to - 27.8)	31.6 (0.3 to 65.8)	70.5 (36.7 to 114.9)	-18.6 (-38.4 to 5.7)	-5.6 (-14.3 to 3.7)	-18.1 (-36.5 to 7.4)	5.5 (-8.8 to 23.2)	-73.9 (-76.0 to - 71.8)	-24.5 (-40.1 to - 4.8)	24.7 (-2.1 to 63.5)
High LDL cholesterol	-6.0 (-15.7 to 5.7)	-43.2 (-51.7 to - 33.4)	-17.2 (-36.1 to 7.0)	22.6 (0.6 to 50.1)	-38.2 (-52.9 to -15.3)	-16.9 (-22.5 to - 9.6)	-37.7 (-53.3 to - 18.3)	-1.1 (-16.8 to 15.8)	-74.5 (-75.9 to - 73.0)	-34.8 (-48.5 to - 18.8)	7.4 (-16.3 to 37.2)
High systolic blood pressure	-11.0 (-21.5 to 2.3)	-51.0 (-58.9 to - 40.7)	-27.7 (-45.4 to - 6.4)	10.0 (-9.7 to 32.7)	-36.8 (-52.9 to -16.2)	-18.2 (-24.0 to - 12.2)	-36.3 (-50.8 to - 15.1)	-4.7 (-20.3 to 13.3)	-79.3 (-82.1 to - 76.5)	-34.1 (-48.4 to - 16.6)	-1.8 (-24.8 to 24.9)
Kidney dysfunction	-12.7 (-22.8 to -2.2)	-49.2 (-56.1 to - 40.6)	-21.9 (-38.3 to - 2.1)	11.6 (-8.6 to 34.0)	-38.0 (-52.3 to -18.2)	-25.7 (-30.9 to - 20.3)	-39.5 (-53.9 to - 19.1)	-3.9 (-17.6 to 12.1)	-75.4 (-77.7 to - 73.5)	-36.2 (-48.9 to - 20.6)	-11.2 (-31.2 to 13.5)
Low physical activity	6.3 (-15.2 to 32.0)	-49.3 (-65.6 to - 26.2)	-12.3 (-54.3 to 52.0)	24.7 (-6.6 to 61.4)	-36.2 (-63.6 to -0.4)	-12.5 (-38.4 to 25.4)	-29.0 (-60.8 to 14.4)	0.7 (-34.2 to 49.9)	-77.4 (-84.0 to - 66.8)	-39.3 (-59.3 to - 4.9)	13.0 (-29.5 to 91.4)
Non-optimal temperature	-6.5 (-22.7 to 29.0)	-44.5 (-62.4 to - 20.6)	19.8 (-158.3 to 167.5)	3.6 (-23.5 to 69.7)	-24.5 (-43.7 to 4.8)	-5.1 (-19.1 to 53.1)	-35.0 (-50.7 to - 7.6)	114.9 (-1296.8 to 582.7)	-55.3 (-78.1 to 5.6)	11.2 (-29.6 to 85.5)	16.4 (-11.9 to 66.5)
Other environmental risks	-21.9 (-32.4 to -8.0)	-56.8 (-63.9 to - 47.9)	-15.7 (-35.2 to 10.1)	-8.5 (-26.7 to 11.7)	-37.0 (-52.5 to -15.1)	-28.8 (-34.7 to - 21.0)	-38.2 (-53.4 to - 16.5)	9.6 (-8.7 to 31.7)	-74.6 (-78.9 to - 71.5)	-38.1 (-52.0 to - 21.9)	-17.4 (-36.9 to 9.0)
Tobacco	-22.5 (-32.3 to -10.7)	-63.1 (-70.2 to - 54.6)	-22.7 (-41.3 to - 0.4)	12.1 (-10.4 to 39.4)	-40.5 (-55.2 to -21.0)	-36.7 (-42.8 to - 29.6)	-63.4 (-73.6 to - 50.1)	-25.4 (-39.5 to - 7.8)	-78.8 (-81.2 to - 76.2)	-48.1 (-60.1 to - 33.9)	-16.6 (-37.5 to 11.6)

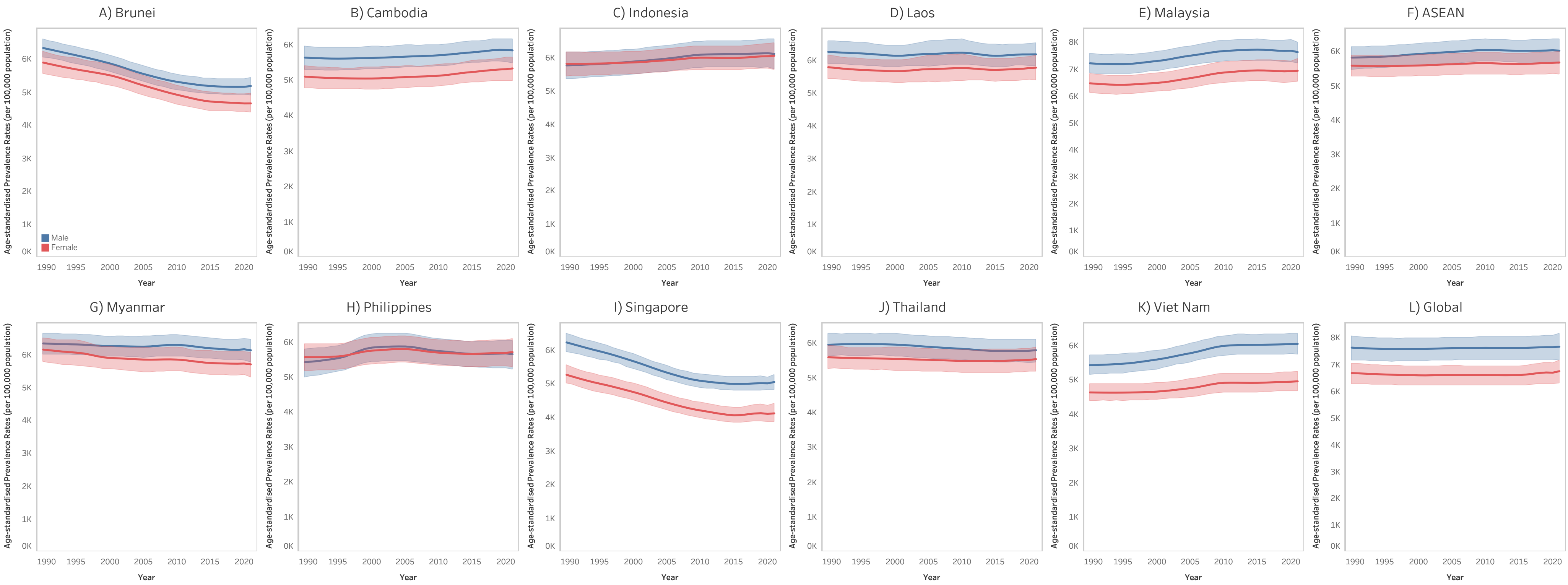

Figure S1: Trends in age-standardised prevalence rates (per 100 000 population) from 1990 to 2021 across global, ASEAN and ASEAN countries by sex

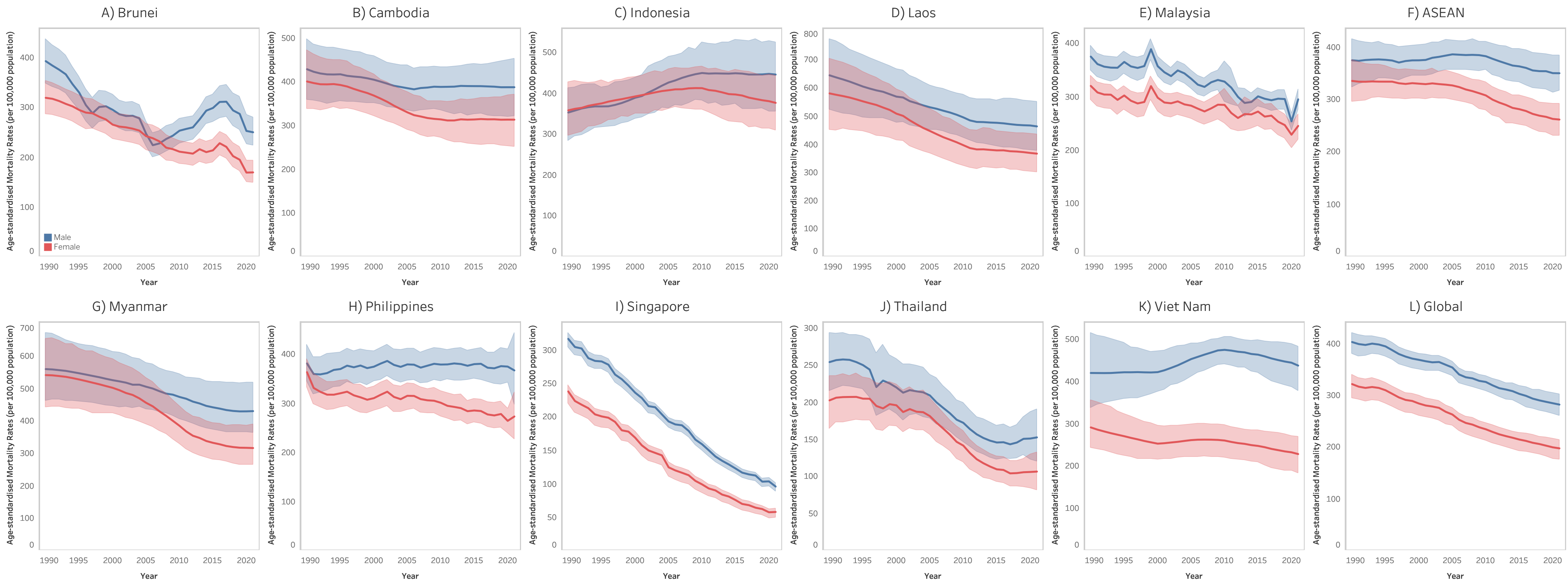

Figure S2: Trends in age-standardised mortality rates (per 100 000 population) from 1990 to 2021 across global, ASEAN and ASEAN countries by sex

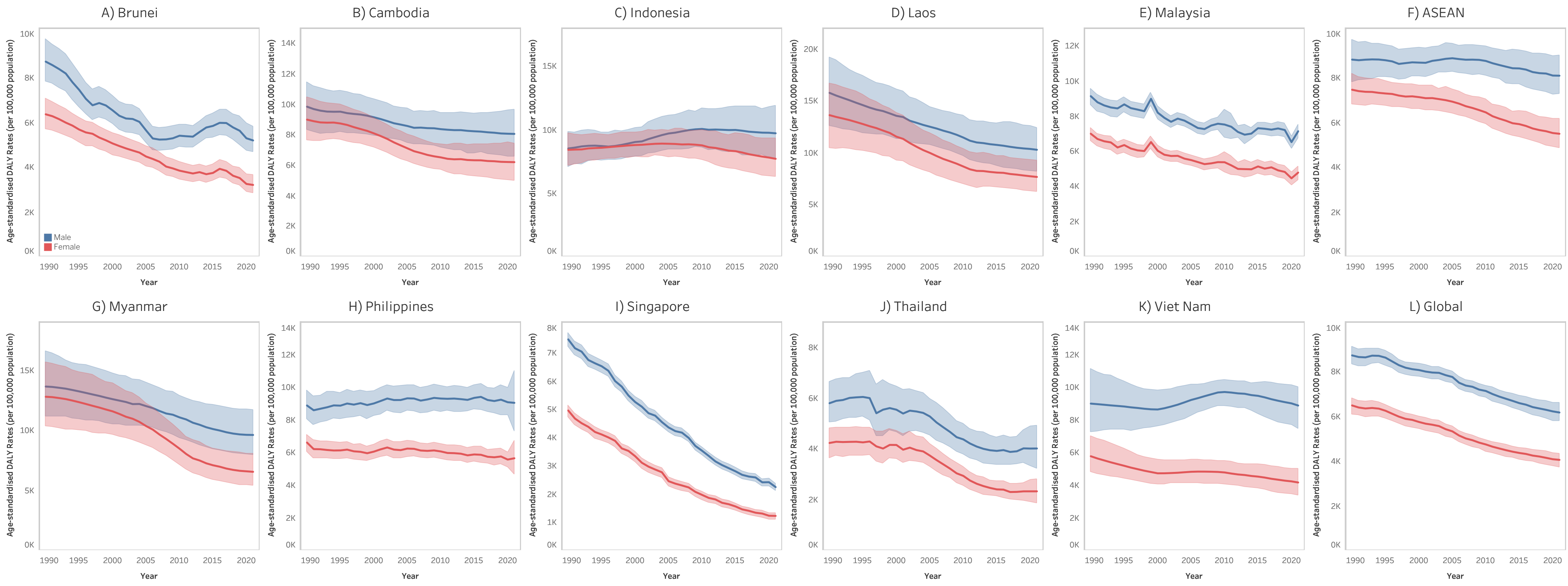

Figure S3: Trends in age-standardised DALY rates (per 100 000 population) from 1990 to 2021 across global, ASEAN and ASEAN countries by sex

Figure S4: Age-distribution of number of CVD (A) prevalence cases, (B) number of mortality, (C) number of DALYs across ASEAN

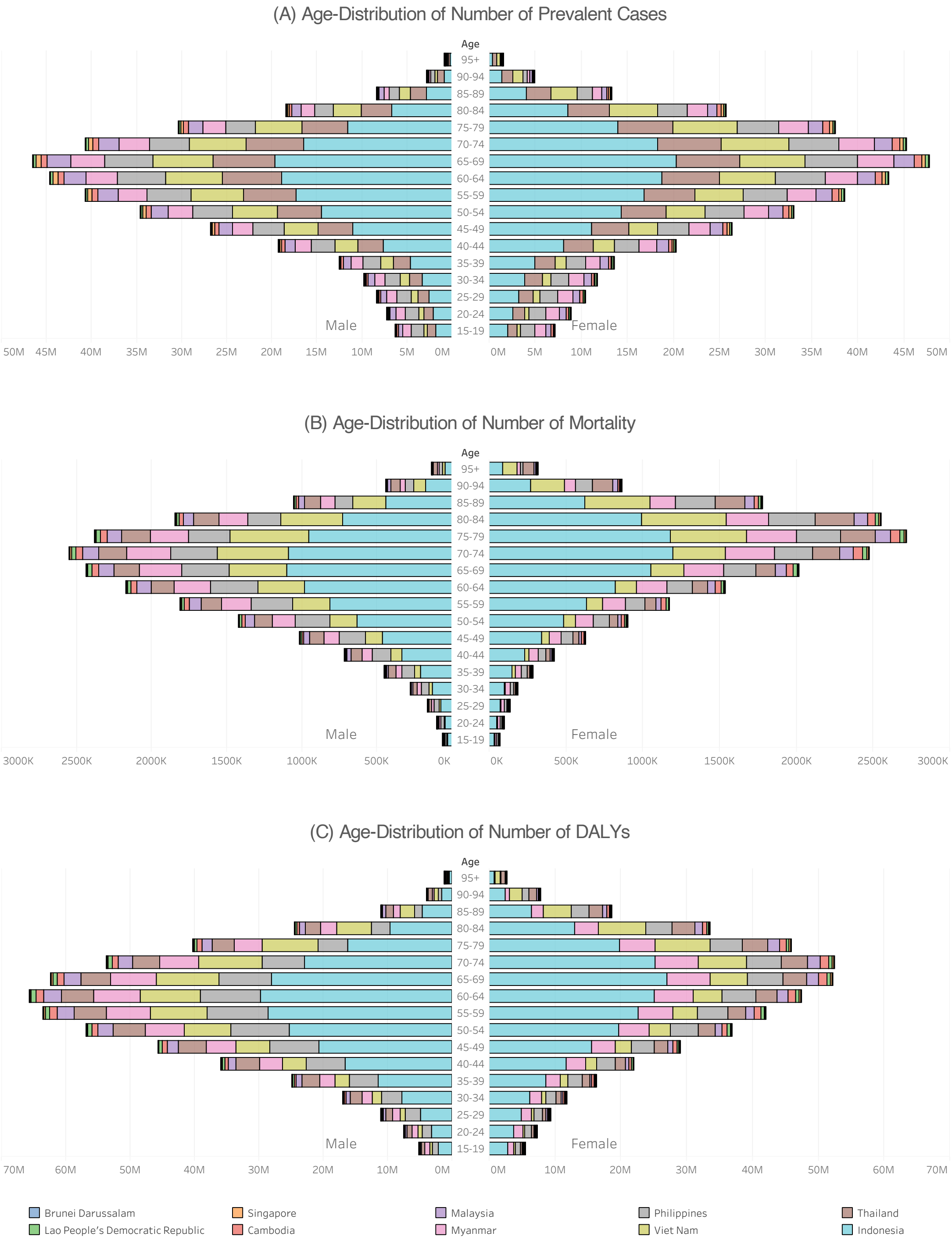

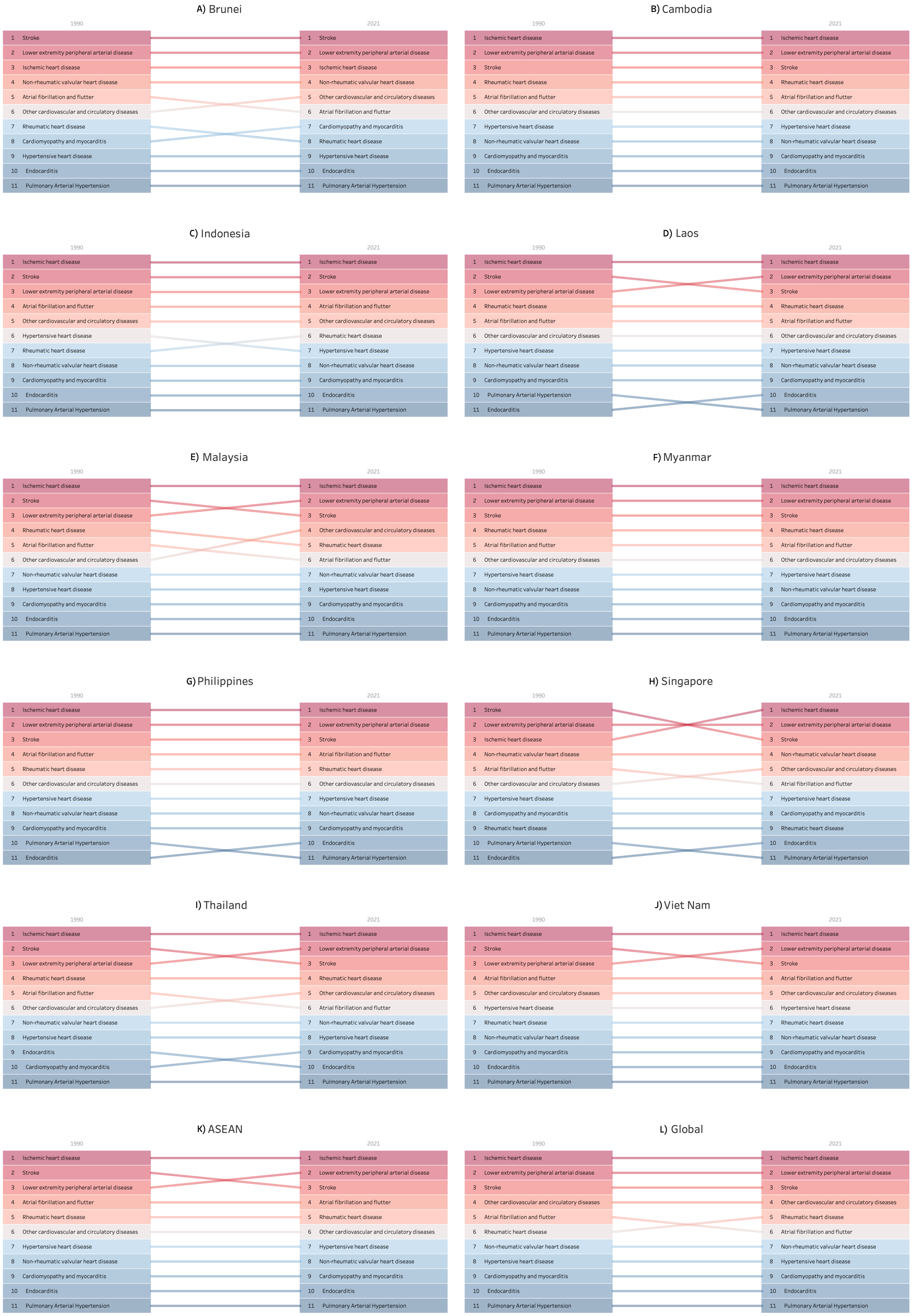

Figure S5: Ranking of cardiovascular diseases causes based on age-standardised prevalence rates (per 100 000 population) from 1990 to 2021 by global, ASEAN and ASEAN countries

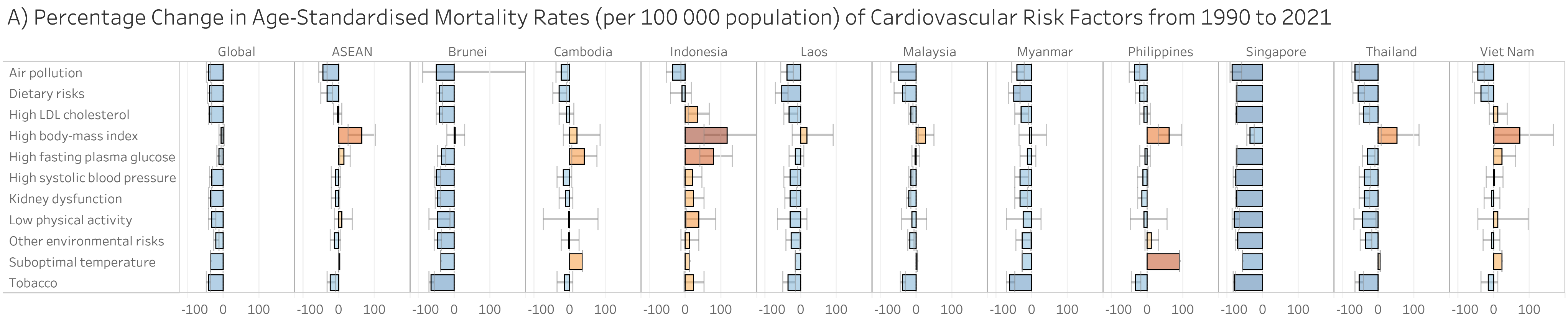

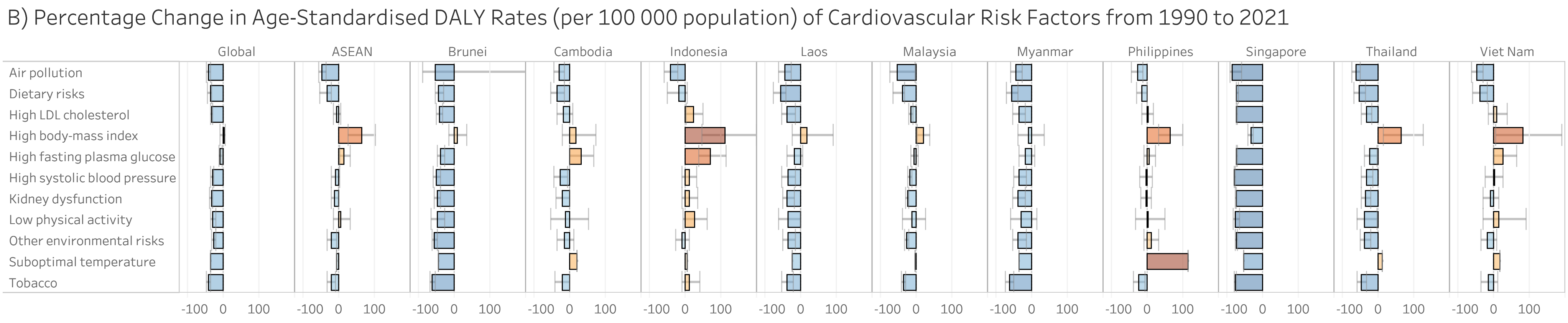

Figure S6: Percentage change in (A) age-standardised mortality rates and (B) age-standardised DALY rates (per 100,000 population) of CVD risk factors from 1990 to 2021 by global, ASEAN and ASEAN countries

References

1 Johnson SC, Cunningham M, Dippenaar IN, *et al.* Public health utility of cause of death data: applying empirical algorithms to improve data quality. *BMC Medical Informatics and Decision Making* 2021; **21**: 175.

2 GBD 2021 Causes of Death Collaborators. Global burden of 288 causes of death and life expectancy decomposition in 204 countries and territories and 811 subnational locations, 1990-2021: a systematic analysis for the Global Burden of Disease Study 2021. *Lancet* 2024; **403**: 2100–32.

3 GBD 2021 Diseases and Injuries Collaborators. Global incidence, prevalence, years lived with disability (YLDs), disability-adjusted life-years (DALYs), and healthy life expectancy (HALE) for 371 diseases and injuries in 204 countries and territories and 811 subnational locations, 1990-2021: a systematic analysis for the Global Burden of Disease Study 2021. *Lancet* 2024; **403**: 2133–61.

4 Fullman N, Yearwood J, Abay SM, *et al.* Measuring performance on the Healthcare Access and Quality Index for 195 countries and territories and selected subnational locations: a systematic analysis from the Global Burden of Disease Study 2016. *The Lancet* 2018; **391**: 2236–71.

5 Global Burden of Disease Study 2021 (GBD 2021) Socio-Demographic Index (SDI) 1950–2021. 2024. https://ghdx.healthdata.org/record/global-burden-disease-study-2021-gbd-2021-socio-demographic-index-sdi-1950%E2%80%932021 (accessed Feb 18, 2025).

6 Mensah GA, Fuster V, Murray CJL, Roth GA. Global Burden of Cardiovascular Diseases and Risks, 1990-2022. *J Am Coll Cardiol* 2023; **82**: 2350–473.

Authors' affiliations

Yong Loo Lin School of Medicine (L H Goh PhD, J Jayabaskaran MBBS, S Nagarajan MBBS, Prof N Venketasubramanian MSc, N W Chew MD, M Ng PhD), Department of Medicine (B Chong MBBS), Division of Family Medicine (S C C van der Lubbe PhD), Cardiovascular Metabolic Translational Research Program (M Dalakoti MPH), Saw Swee Hock School of Public Health (S Ramazanu PhD), Centre for Research in Health Systems Performance (Prof J Valderas PhD), National University of Singapore, Singapore; Department of Cardiology (J Chia BSc), National University Health System, Singapore; Institute for Health Metrics and Evaluation (C O Johnson PhD, X Dai PhD, C Bisignano MPH, Prof S I Hay FMedSci, Prof C J L Murray DPhil, G A Roth MD, M Ng PhD), Department of Health Metrics Sciences, School of Medicine (X Dai PhD, Prof S I Hay FMedSci, Prof C J L Murray DPhil, G A Roth MD), Division of Cardiology (G A Roth MD), University of Washington, Seattle, WA, USA; Faculty of Medicine and Public Health (B Aji DrPH), Jenderal Soedirman University, Purwokerto, Indonesia; Department of Medicine (K T Aldecoa MD), Trinity Health Oakland Hospital/Wayne State University, Pontiac, MI, USA; Wayne State University, Detroit, MI, USA (K T Aldecoa MD); Department of Public Health and Community Medicine (Prof S M Aljunid PhD), International Medical University, Kuala Lumpur, Malaysia; International Centre for Casemix and Clinical Coding (Prof S M Aljunid PhD), National University of Malaysia, Bandar Tun Razak, Malaysia; Department of General Medicine (R A Ananda MD), Eastern Health, Box Hill, VIC, Australia; School of Medicine and Public Health (G C Apostol MD), Center for Research and Innovation (V F Pepito MSc), Ateneo De Manila University, Pasig City, Philippines; Inter-Agency Committee on Environmental Health (G C Apostol MD), Health Technology Assessment Unit (Y H Zuniga BS), Department of Health Philippines, Manila, Philippines; Department of Paediatrics (Prof H Ariffin PhD), University of Malaya Medical Centre (Prof H Ariffin PhD), Department of Primary Care Medicine (J Jamaluddin MMed), University of Malaya, Kuala Lumpur, Malaysia; Nursing Department (Y Asri PhD), Faculty of Health Science (Y Asri PhD), Institute of Technology and Health Science RS dr Soepraoen, Malang, Indonesia; International Medical School (A A Baig PhD), Management and Science University, Alam, Malaysia; Department of Epidemiology and Biostatistics (A C Bermudez MD), Department of Health Policy and Administration (E A A Faraon MD), Department of Health Policy and Administration, College of Public Health (Prof F B Garcia PhD), University of the Philippines Manila, Manila, Philippines; Department of Epidemiology (A C Bermudez MD), Brown University, Providence, RI, USA; College of Public Health, Medical, and Veterinary Sciences (M Cenderadewi MPHTM), James Cook University, Townsville, QLD, Australia; Department of Public Health (M Cenderadewi MPHTM), University of Mataram, Mataram, Indonesia; Faculty of Humanities and Health Sciences (H Chen MSc), Curtin University, Miri, Malaysia; Department of Public Health and Primary Care (M Dalakoti MPH), University of Cambridge, Cambridge, UK; Advanced Nursing Department (F Efendi PhD), Department

of Epidemiology Population Biostatistics and Health Promotion (A Hargono PhD), Department of Advanced Nursing (E M M Has PhD), Special Research Taskforce for Indonesia Healthcare System (F R Muharram MD), Universitas Airlangga (Airlangga University), Surabaya, Indonesia; Centre for Public Health, Equity and Human Flourishing (N K Fauk PhD), Torrens University Australia, Adelaide, SA, Australia; Institute of Resource Governance and Social Change, Kupang, Indonesia (N K Fauk PhD); School of Nursing and Midwivery (E M M Has PhD), La Trobe University, Bundoora, VIC, Australia; Faculty of Nursing (F Hasan PhD, D S Romadlon PhD), Center of Excellence in Genomics and Precision Dentistry (T Porntaveetus PhD), Chulalongkorn University, Bangkok, Thailand; Faculty of Pharmacy (U I Ibrahim PhD), Sultan Zainal Abidin University, Malaysia, Terengganu, Malaysia; Faculty of Public Health (M Iqhrammullah PhD), Universitas Muhammadiyah Aceh, Banda Aceh, Indonesia; School of Pharmacy (B Iskandar PhD), School of Nursing (M Kurniasari PhD, A L Wicaksana MS), Taipei Medical University, Taipei, Taiwan; Department of Pharmaceutical Technology (B Iskandar PhD), Sekolah Tinggi Ilmu Farmasi Riau, Pekanbaru, Indonesia; Department of Clinical Pharmacy & Pharmacy Practice (Prof N Ismail PhD), Asian Institute of Medicine, Science and Technology, Bedong, Malaysia; Malaysian Academy of Pharmacy, Puchong, Malaysia (Prof N Ismail PhD); Rothschild Foundation Hospital (Prof J B Jonas MD), Institut Français de Myopie, Paris, France; Singapore Eye Research Institute (Prof J B Jonas MD), Singapore Eye Research Institute, Singapore, Singapore; NIHR Global Health Research Unit on Global Surgery (S K Kamarajah MD), University of Birmingham, Birmingham, UK; School of Traditional Chinese Medicine (Y Kim PhD), Xiamen University Malaysia, Sepang, Malaysia; Faculty of Medicine and Health Science (M Kurniasari PhD), Universitas Kristen Satya Wacana, Salatiga, Indonesia; National Research and Innovation Agency (BRIN), Jakarta, Indonesia (A Kusnali MA); Institute for Health Sciences (C Y Kustanti PhD), STIKES Bethesda Yakkum Yogyakarta Indonesia, Yogyakarta, Indonesia; School of Digital Science (D Lai PhD), Institute of Applied Data Analytics (D Lai PhD), Institute of Health Sciences (S Ong FAMS), Universiti Brunei Darussalam (University of Brunei Darussalam), Bandar Seri Begawan, Brunei; Department of Obstetrics and Gynecology (G A Lukas MD), University of Indonesia, Jakarta, Indonesia; Indonesian Public Health Association, (G A Lukas MD); Centre for Public Health and Wellbeing (Z Ma PhD), University of the West of England, Bristol, UK; Universiti Brunei Darussalam (University of Brunei Darussalam), (Z Ma PhD); Faculty of Public Health (Prof S Martini PhD), Universitas Airlangga (University of Airlangga), Surabaya, Indonesia; Indonesian Public Health Association, Surabaya, Indonesia (Prof S Martini PhD); Faculty of Humanities and Health Sciences (Prof R R Marzo MD), Curtin University, Sarawak, Malaysia; Jeffrey Cheah School of Medicine and Health Sciences (Prof R R Marzo MD), Monash University, Subang Jaya, Malaysia; International PhD Program in Biotech and Healthcare Management (S Melisa MBA), Taipei Medical University, New Taipei City, Taiwan; School of Medical Sciences (Prof K Musa PhD), Science University of Malaysia, Kubang Kerian, Malaysia; Faculty of Pharmacy (Prof F

Nainu PhD), Hasanuddin University, Makassar, Indonesia; National Dental Research Institute Singapore (G G Nascimento PhD), Duke-NUS Medical School, Singapore, Singapore; Department of Non-communicable Disease Epidemiology (A Nur MPH), London School of Hygiene & Tropical Medicine, London, UK; Center for Health System and Strategy (A Nur MPH), Ministry of Health, Jakarta, Indonesia; Department of Public Health (S Ong FAMS), Ministry of Health, Bandar Seri Begawan, Brunei; Health Sciences Department (D R A Pribadi MSc), Muhammadiyah University of Surakarta, Sukoharjo, Indonesia; Department of Nutrition Science (S Rahmawaty PhD), Muhammadiyah University of Surakarta, Surakarta, Indonesia; Department of Midwifery (K Ramadhan MPH), Ministry of Health of the Republic of Indonesia, Palu, Indonesia; School of Nursing & Health Sciences (S Ramazanu PhD), Hong Kong Metropolitan University, Hong Kong, China; Institute of Epidemiology and Preventive Medicine (Y L Samodra PhD), National Taiwan University, Taipei, Taiwan; Benang Merah Research Center (BMRC), Minahasa Utara, Indonesia (Y L Samodra PhD); Faculty of Dentistry (S Selvaraj PhD), University of Puthisastra, Phnom Penh, Cambodia; Dr. D. Y. Patil Dental College & Hospital (S Selvaraj PhD), Dr. D. Y. Patil Vidyapeeth, Pune (Deemed to be University), Pune, India; Pharmacy Department (C H Setiawan MSc), Sanata Dharma University, Yogyakarta, Indonesia; School of Health Sciences (S Shaharudin PhD), Universiti Sains Malaysia, Kota Bharu, Malaysia; Department of Medical Sciences (Prof V Subramaniyan PhD), Sunway University, Subang Jaya, Malaysia; Faculty of Health Science (D Sulistiyorini MSc), Universitas Indonesia Maju, Jakarta, Indonesia; Department of Biomedical Sciences (Z Sun PhD), Universiti Putra Malaysia, Selangor, Malaysia; National Research and Innovation Agency, Jakarta, Indonesia (I U Tarigan PhD); Faculty of Public Health (J H V Ticoalu MPH), Universitas Sam Ratulangi (Sam Ratulangi University), Manado, Indonesia; Raffles Neuroscience Centre (Prof N Venketasubramanian MSc), Raffles Hospital, Singapore, Singapore; Research Organization for Health (M Wahidin PhD), National Research and Innovation Agency, Bogor, West Java, Indonesia; Department of Medical Surgical Nursing (A L Wicaksana MS), Gadjah Mada University, Yogyakarta, Indonesia; Universitas Sebelas Maret, Surakarta, Indonesia (M A Wijayanto MD); Department of Nursing (A Wilandika PhD), Universitas Aisyiyah Bandung, Bandung, Indonesia; #MentalHealthPH, Inc., Quezon City, Philippines (Y H Zuniga BS).

Authors' contributions

Providing data or critical feedback on data sources

Budi Aji, Kim Abbegail Tan Aldecoa, Syed Mohamed Aljunid, Geminn Louis Carace Apostol, Yuni Asri, Atif Amin Baig, Nicholas WS Chew, Bryan Chong, Xiaochen Dai, Fernando Barroga Garcia, Eka Mishbahatul Marah Has, Faizul Hasan, Benni Iskandar, Nahlah Elkudssiah Ismail, Jayanth Jayabaskaran, Jost B Jonas, Sivesh Kathir Kamarajah, Yun Jin Kim, Maria Dyah Kurniasari, Asep Kusnali, Christina Yeni Yeni Kustanti, Graciella Angelica Lukas, Zheng Feei Ma, Roy Rillera Marzo, Farizal Rizky Muharram, Christopher J L Murray, Kamarul Imran Musa, Aqsha Nur, Sok King Ong, Thantrira Porntaveetus, Sheena Ramazanu, Siddharthan Selvaraj, Vetriselvan Subramaniyan, Narayanaswamy Venketasubramanian, Mugi Wahidin, Matthew Aldo Wijayanto, Angga Wilandika, Yves Miel H Zuniga.

Developing methods or computational machinery

Xiaochen Dai, Simon I Hay, Christopher J L Murray.

Providing critical feedback on methods or results

Budi Aji, Kim Abbegail Tan Aldecoa, Syed Mohamed Aljunid, Roshan A Ananda, Geminn Louis Carace Apostol, Hany Ariffin, Yuni Asri, Atif Amin Baig, Amiel Nazer C Bermudez, Muthia Cenderadewi, Hana Chen, Nicholas WS Chew, Bryan Chong, Xiaochen Dai, Mayank Dalakoti, Ferry Efendi, Emerito Jose A Aquino Faraon, Fernando Barroga Garcia, Lay Hoon Goh, Arief Hargono, Eka Mishbahatul Marah Has, Faizul Hasan, Simon I Hay, Umar Idris Ibrahim, Muhammad Iqhrammullah, Benni Iskandar, Nahlah Elkudssiah Ismail, Jayanth Jayabaskaran, Catherine O Johnson, Jost B Jonas, Sivesh Kathir Kamarajah, Yun Jin Kim, Maria Dyah Kurniasari, Asep Kusnali, Christina Yeni Yeni Kustanti, Daphne Teck Ching Lai, Graciella Angelica Lukas, Zheng Feei Ma, Santi Martini, Roy Rillera Marzo, Septi Melisa, Farizal Rizky Muharram, Christopher J L Murray, Kamarul Imran Musa, Firzan Nainu, Marie Ng, Aqsha Nur, Thantrira Porntaveetus, Dimas Ria Angga Pribadi, Setyaningrum Rahmawaty, Kadar Ramadhan, Debby Syahru Romadlon, Yoseph Leonardo Samodra, Siddharthan Selvaraj, Christianus Heru Setiawan, Shazlin Shaharudin, Vetriselvan Subramaniyan, Desy Sulistiyorini, Zhong Sun, Ingan Ukur Tarigan, Jansje Henny Vera Ticoalu, Jose M Valderas, Narayanaswamy Venketasubramanian, Mugi Wahidin, Anggi Lukman Wicaksana, Matthew Aldo Wijayanto, Angga Wilandika, Yves Miel H Zuniga.

Drafting the work or revising it critically for important intellectual content

Kim Abbegail Tan Aldecoa, Roshan A Ananda, Geminn Louis Carace Apostol, Hany Ariffin, Yuni Asri, Atif Amin Baig, Catherine Bisignano, Muthia Cenderadewi, Hana Chen, Nicholas WS Chew, Jobelle Chia, Bryan Chong, Mayank Dalakoti, Ferry Efendi, Emerito Jose A Aquino Faraon, Nelsensius Klau Fauk, Lay Hoon Goh, Eka Mishbahatul Marah Has, Faizul Hasan, Simon I Hay, Umar Idris Ibrahim, Muhammad Iqhrammullah, Nahlah

Elkudssiah Ismail, Jazlan Jamaluddin, Jayanth Jayabaskaran, Catherine O Johnson, Jost B Jonas, Sivesh Kathir Kamarajah, Yun Jin Kim, Maria Dyah Kurniasari, Christina Yeni Yeni Kustanti, Daphne Teck Ching Lai, Graciella Angelica Lukas, Zheng Feei Ma, Roy Rillera Marzo, Septi Melisa, Farizal Rizky Muharram, Christopher J L Murray, Kamarul Imran Musa, Gustavo G Nascimento, Marie Ng, Aqsha Nur, Veincent Christian Filipino Pepito, Thantrira Porntaveetus, Kadar Ramadhan, Debby Syahru Romadlon, Gregory A Roth, Siddharthan Selvaraj, Shazlin Shaharudin, Jose M Valderas, Stephanie C. C. van der Lubbe, Narayanaswamy Venketasubramanian, Mugi Wahidin, Anggi Lukman Wicaksana.

Managing the estimation or publications process

Simon I Hay, Christopher J L Murray, Marie Ng, Gregory A Roth.
